# *Guanchochroma wildpretii* gen. et spec. nov. (Ochrophyta) Provides New Insights into the Diversification and Evolution of the Algal Class Synchromophyceae

**DOI:** 10.1371/journal.pone.0131821

**Published:** 2015-07-02

**Authors:** Maria Schmidt, Susanne Horn, Katrin Ehlers, Christian Wilhelm, Reinhard Schnetter

**Affiliations:** 1 Universität Leipzig, Department of Plant Physiology, Johannisallee 21–23, 04103 Leipzig, Germany; 2 Universitätsklinikum Essen, Klinik für Dermatologie, Forschungslabor, Hufelandstr. 55, 45447 Essen, Germany; 3 Justus-Liebig-Universität Gießen, Institut für Botanik, Heinrich-Buff-Ring 38, 35392 Giessen, Germany; University of Innsbruck, AUSTRIA

## Abstract

A new relative of the chrysophyte genus *Chrysopodocystis* was found in Tenerife and termed *Guanchochroma wildpretii*. This unicellular alga was most noticeably discernible from *Chrysopodocystis socialis* (the only species of this genus) by the presence of a cyst-like stage with a multilayered lorica, which also functions as a dispersal unit and shows secondary wall growth. Secondary expansion of loricae (cell casings not involved in cell division, usually with a more or less pronounced opening) has never been observed previously and marks a unique feature of the new taxon. Plastids are non-randomly distributed within cells of *G*. *wildpretii*. 18S rRNA gene analyses identified the two species as sister lineages and placed them in a monophyletic group with the Synchromophyceae, a heterokont algal (Ochrophyta) class characterized by the presence of chloroplast complexes. Yet, neither *Chrysopodocystis* nor *Guanchochroma* showed this feature in ultrastructure analyses. Additionally, their 18S rRNA genes possessed distinct inserts, the highest GC-content known for Ochrophyta and exceptionally long branches on the Ochrophyta 18S rDNA phylogenetic tree, suggesting substantially increased substitution rates along their branch compared to Synchromophyceae. Plastid marker data (*rbc*L) recovered a monophyletic clade of *Chrysopodocystis*, *Guanchochroma* and Synchromophyceae as well, yet with lower supports for internal split order due to limited resolution of the marker. Evidence for the sequence of events leading to the formation of the plastid complex of Synchromophyceae still remains ambiguous because of the apparently short timeframe in which they occurred.

## Introduction

**Fig 1:**
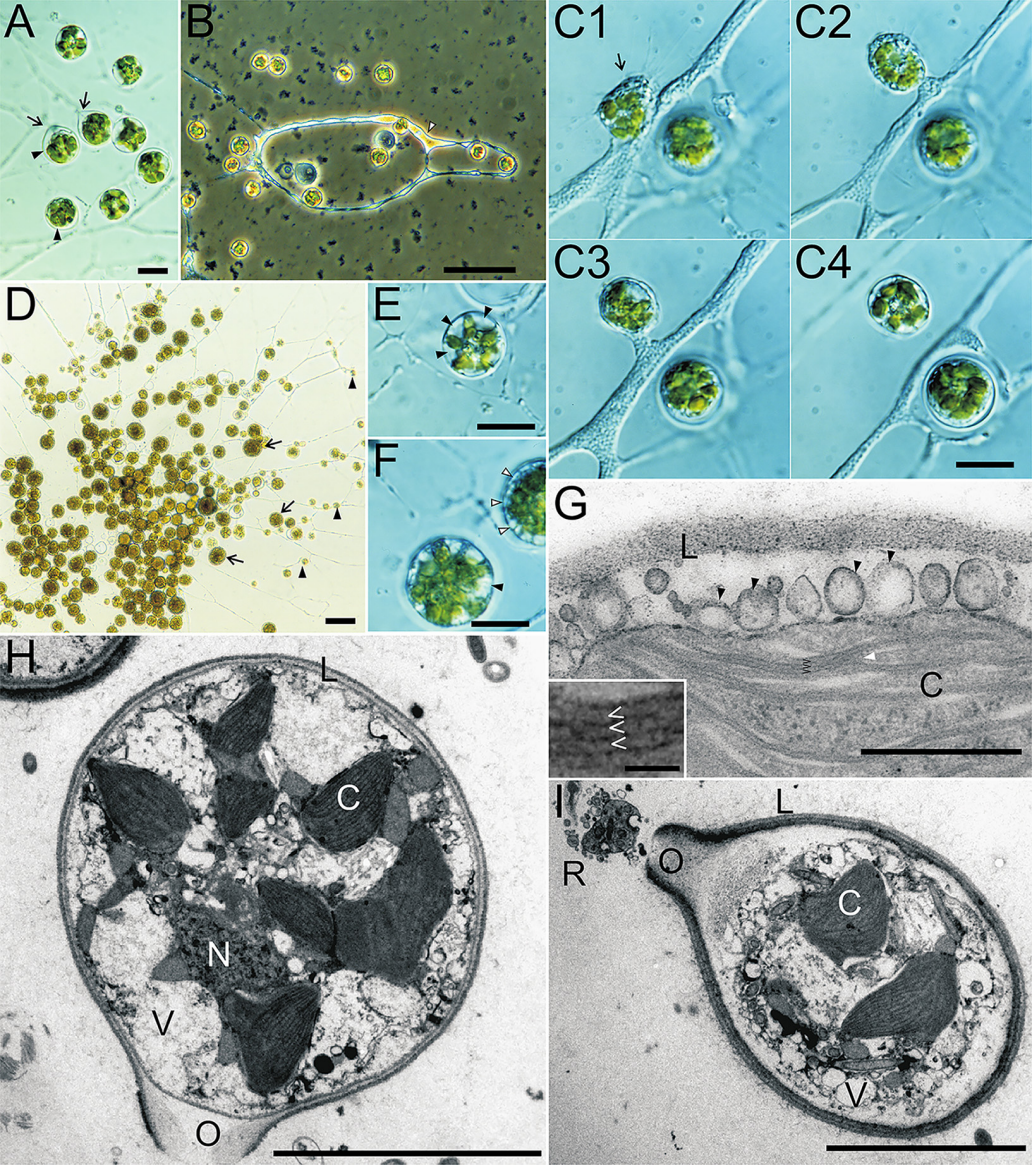
Morphology of *Guanchochroma wildpretii*. (A) MCBs of young amoebae of *G*. *wildpretii* are encased in a thin, hyaline, globular lorica (arrowhead). Reticulopodia exit through the ostiole, which has a short rim (arrow), and fuse to form a meroplasmodium. (B) Migrating amoeba moving in the meroplasmodial network (white arrowhead). (C1–4) A migrating amoeba (arrow) moves on top of the meroplasmodium and settles close to it. Sequence of events from top left to bottom right (10:37 PM, 10:50 PM, 11:49 PM, next day 9:09 PM). (D) Overview of *G*. *wildpretii* colony, comprised of freshly settled cells (arrowhead) and large older cells with multilayered and size-increased loricae (arrow). (E) Freshly settled amoeba with multiple parietal vacuoles (arrowhead) that force the plastids into a star-like shape in the cell center. (F) Sessile cells with multiple vacuoles (black arrowhead) at the MCB periphery and smaller vesicles (white arrowhead) indicating the position of additional lorica material excretion. (G) TEM of sessile cell with multiple vesicles (arrowhead) at the cell periphery close to the existing lorica. Chloroplast with thylakoids in stacks of three (angle brackets), which can fork (white arrowhead) or pair with other thylakoid stacks. (H) TEM of sessile cell with multilayered lorica, the ostiole of which was closed during the addition of the innermost layer. (I) TEM of sessile cell with a multilayered lorica. A cross section of the reticulopodium is visible in front of the still opened ostiole. C—chloroplast, N—nucleus, O—ostiole, R—reticulopodium, L—lorica, V—vesicle. Scale bars A, C, E,F: 10 μm, B, D: 50 μm, G: 0.5 μm, H, I: 5 μm.

It is commonly accepted that the emergence of chloroplasts (simple plastids with two envelope membranes) can be traced back to a singular endocytobiotic event involving a cyanobacterium and a heterotrophic eukaryote [1], although exceptions are possible (see [2], *Paulinella chromatophora*). Yet organisms with complex plastids (surrounded by three or four envelope membranes) have evolved multiple times via endocytobioses, where green and red algae, or even organisms with complex plastids themselves, were engulfed by heterotrophic members of some eukaryotic supergroups (Rhizaria, Alveolata, Stramenopiles, CCTH clade and Excavata) and retained as organelles (see [3], Fig 1). The number of such independently occurring plastid transfers is under constant scientific debate (e.g. [4–7]).

Since the advent of DNA sequence based phylogenies, protists that were previously described based on morphology alone could be assigned to existing taxonomic groups (e.g. [8]). This holds true especially for cases in which, because of pronounced radiation events, the morphology is heavily divergent from the description of the taxonomic group it belongs to, or the above-mentioned transfer of chloroplasts among lineages obscures the true origin. One previously solitary, unmated organism is the non-photosynthetic amoeba *Leukarachnion* sp. PRA-24, which was found to be a close relative of the Synchromophyceae [8], a new class of heterokont algae (Ochrophyta or stramenopiles). This class was predominantly established based on a unique plastid morphology feature called the plastid complex. This complex differs from general heterokont plastid morphology in its membrane ultrastructure, i.e. 5–8 single plastids share a common outer membrane pair (periplastid membrane/PM and epiplastid rough endoplasmic reticulum/EPrER) instead of just the outermost membrane [9]. Even though this key feature of the Synchromophyceae is missing in *Leukarachnion* sp. PRA-24, 18S rDNA sequences consistently recovered it close to the two known Synchromophyceae species, *Synchroma grande* and *Synchroma pusillum*, together with another photosynthetic amoeba lacking the chloroplast complex, *Chlamydomyxa labyrinthuloides* [8,10,11]. Taken together, this creates a morphologically variable clade with highly divergent, amoeboid genera, for which the only common morphological trait that can be readily observed is the meroplasmodium (see Table 1), i.e. the fusion of reticulopodia with the retention of individual main cell bodies (MCBs). Koch et al. [12] already noted that the combination of life cycle stages and strategies of the Synchromophyceae may promote diversification processes in this taxon.

**Table 1:** Characteristic features of Synchromophyceae and related amoeboid algae. Features are taken from [8,9,11,14,68] and this study. NAO—North Atlantic Ocean, MS—Mediterranean Sea, CS—Caribbean Sea, EC- English Channel.

	*Leukarachnion* sp. RA-24	*Ch*. *labyrinthuloides*	*C*. *socialis*	*G*. *wildpretii*	*S*. *grande*	*S*. *pusillum*
plastid(s)	no	yes	yes	yes	yes	yes
plastid number	-	7–10 (monoenergide cells)	~ 20	initially 5–8	6–8 (per complex)	6–8 (per complex)
plastid complex	-	no	no	no	yes	yes
girdle lamella	-	yes	no	no	no	no
capping vesicle	-	no	no	no	yes	yes
pyrenoids	-	no	no	no	yes	yes
different cell stages	yes	yes	yes	yes	yes	yes
flagellate cells	no	no	no	no	no	no
sessile amoebae	no	yes	yes	yes	yes	yes
lorica	no	no	globular	globular	flattened	flattened
lorica pore (ostiole)	-	-	lateral	lateral	lateral	lateral
lorica with sec. growth	-	-	no	yes	no	no
walled amoebae (cysts)	yes	yes	yes	yes	yes	yes
migrating amoebae (MA)	yes	yes	yes	yes	yes	yes
floating amoebae (FA)	?	no	yes	no	yes	yes
meroplasmodium	yes	yes	yes	yes	yes	yes
main dispersal unit	?	cyst	MA/FA	cysts-like stage	MA/FA	MA/FA
lorica diameter (μm)	-	-	12–13	10–12	26	8–12
multinucleate cells	yes	yes	no	no	yes*	yes*
binary division	?	yes	yes	yes	yes	yes
isolate location(s)	NAO	Austria	EC	NAO	NAO, MS, CS	NAO, CS
habitat	salt marsh	limnic	marine	marine	marine	marine

* Under culture conditions multiple amoebae (>2) fusion was observed, forming large migrating/floating amoebae. It could not be determined whether these are cell aggregates (ectoplasmic fusion only) or fusion plasmodia (with endoplasmic fusion).

*S*. *grande* and *S*. *pusillum* are morphologically almost identical and only differ in lorica diameter and the number of chloroplast complexes per cell [11]. The three cell types found in the two *Synchroma* species are floating (heliozoan-like), migrating and sessile amoebae. The sessile amoeba is encased in a hyaline, flattened lorica, which holds the MCB and connects to the meroplasmodium. In contrast to a cell wall, the lorica is a cell casing which is never involved in cell division, more or less opened to one side (in case of *Synchroma* with a small aperture called ostiole) and can be attached to a substrate or other loricae (colony formation) [13]. The other two cell types are naked, solitary amoebae, which arise by hatching from the lorica. They are interconvertible and either float freely in the medium or migrate on substrates, respectively.

Another organism of interest here is *Chrysopodocystis socialis*, described in 1978 by Billard [14] as an amoeboid, colony-forming alga whose MCBs are encased in hyaline loricae. *C*. *socialis* was characterized as a member of the family Stylococcaceae (order Rhizochrysidales, class Chrysophyceae, Ochrophyta) according to its morphological features alone, especially the similarity to the *Heliapsis* species, *H*. *achromatica* and *H*. *mutabilis* [14]. Interestingly, this family currently includes many monotypic genera [15] with highly variable “body plans”. A noted similarity of the Rhizochrysidales is the alternation between a colonial, rhizopodial organization and an amoeboid, non-flagellate phase [14]. Since this feature, as stated above, is also present in the Synchromophyceae, a sister class to the Chrysophyceae, and no sequence data are available for *C*. *socialis*, this species is included in the present study, especially since the roughly 20 plastids per cell are apparently not grouped into plastid complexes [14]. Differences to *Synchroma* are found, e.g. in the lorica shape, the parietal chloroplast positioning and the lack of pyrenoids.

In this study we aim to elucidate the position of the chrysophyte *C*. *socialis*, which shares several features with the *Synchroma* species, but, due to the absence of a plastid complex and its globular lorica, is distinct from them (see Table 2 in [9]). Additionally, we establish a new genus and species, *Guanchochroma wildpretii*, which was collected in Tenerife. *G*. *wildpretii* can be morphologically placed into the same group of loricate, network-forming algae and can provide insight into the phylogeny of *C*. *socialis* and the Synchromophyceae. In addition to morphological assessments, we use a range of phylogenetic tree construction methods to infer the position of the newly described species relative to *C*. *socialis*, the Synchromophyceae, *Chlamydomyxa* and *Leukarachnion*.

## Methods

### Algae isolation and cultivation

Environmental material from a benthic, sublittoral habitat was collected by R. Schnetter in November 2009 in Tenerife, at a collection site situated between the Roca Negra and the light house at Punta del Hidalgo (Canary Islands, Spain). Unialgal cultures of *G*. *wildpretii* were obtained from this material by R. Schnetter and cultivated in Petri dishes under periodic microscopical observation as described in [11]. Type material was deposited at the Roscoff Culture Collection under Accession number RCC3390. The sample including *G*. *wildpretii* was collected at a public, openly accessible site without environmental protective regulations, and R. Schnetter did not require specific permission to retrieve or transfer it within the EU. Additionally, an agreement with the Universidad de La Laguna (Tenerife, Spain) related to such field work exists. The holotype of the new species was transferred to the herbarium of the Universidad de La Laguna in fulfilment of this agreement. *Chrysopodocystis socialis* (isolated by C. Billard in 1972 and used for description of the species in 1978), was obtained from Algobank-Caen as AC38 and cultured in von Stosch’s enriched seawater medium [16]. *Chlamydomyxa labyrinthuloides* P42150 was obtained from the Culture Collection of Algae Marburg (CCAM) and cultured in Petri dishes with Gleodinium-Lsg. II (CCAM) according to von Stosch.

### Light microscopy and TEM

Light microscopy images and transmission electron images of *C*. *socialis* and *G*. *wildpretii* were obtained and processed as described by [9]. A modified illumination system and cultures in plastic Petri dishes with cover glasses (0.17 mm thick) inserted into bottom and top allowed for DIC photographs of living material. An additional Leica NPL Fluotar 100/1.20 W FLUORESZENZ objective in combination with an inverted FU microscope was used. TEM digital images were obtained with the LEO 912 Omega (Carl Zeiss AG, Germany).

### DNA extraction, PCR and sequencing, pigment analysis

Cells of *Ch*. *labyrinthuloides*, *C*. *socialis* and *G*. *wildpretii* were collected by scraping biofilm off the Petri dish surface and pelleting at 2500xg for 5 minutes. DNA was extracted from fresh or frozen cell pellets using either the InnuPREP Plant DNA Kit (Analytic Jena), a CTAB extraction protocol with a subsequent phenol-chloroform-purification [17] or the DNeasy Plant Mini Kit (Qiagen). For gene amplification, the Peqlab Taq-DNA-Polymerase all inclusive (Erlangen, Germany) was used according to the manufacturer’s instructions with *rbc*L and 18S primers listed in [11]. PCR fragments were cloned into the pCR4-TOPO vector using the TA Cloning Kit for sequencing (Invitrogen), and at least 4 clones were sequenced by GATC Biotech (Konstanz, Germany) using standard primers. DNA extraction, PCR, cloning, and sequencing were replicated for each species at least twice. Consensus sequences were deposited in Genbank under accessions KF443034—KF443039.

Cells of *G*. *wildpretii* were harvested as stated above, flash frozen in liquid nitrogen and HPLC pigment analysis carried out as described in [18] in triplicate, with identification of pigments according to authentic standards.

### DNA marker analysis

For 18S GC-content and length calculations, all eukaryotic sequences with >1500 nt length and >90% quality were retrieved from the SILVA database [19] on 17-Mar-2013 (20277 entries total) with common gaps. This dataset and sequences obtained in this study were cut to the homologous region available for *S*. *grande* CCMP2876 (DQ788730), with entries not covering this region removed and all remaining entries degapped. These 17090 entries (including 1098 stramenopiles) were processed for visualization of GC-content and length using R3.0.0 [20] with the additional package seqinr3.0–7 [21]. Variable region 8 secondary structure analysis of the 18S gene in selected taxa was calculated using the Mfold web server [22], with manual refinement and visualization carried out under VARNA [23], guided by the rRNA secondary structure model with eukaryotic helix numbering according to [24] and definition of variable regions with positions of non-universal helices given in [25]. Helix E46 sequence comparison was carried out in R3.0.0 using the structural eukaryotic alignment mentioned above without degapping.

For 18S rDNA marker analysis, the set of taxa from [11] was obtained from the SILVA database (see S1 Table) as a structural alignment with common gaps and sequences from this study added by the l-ins-I algorithm implemented in mafft 7.023 [26]. After manual refinement, the alignment was treated with gblocks v0.91b [27] under allowance of smaller final blocks and gaps in final blocks present for less than 50% of the dataset, as well as removal of segments with more than 8 contiguous nonconserved positions. The final dataset of 1506 positions contained 78 sequences with 1.18% missing data/gaps. Disparity index calculation included all sites and 1,000 MCMC replicates in MEGA 5.10 [28]. The final 18S dataset analysis of Maximum Likelihood (ML), Neighbor Joining (NJ), and Maximum Parsimony (MP) was calculated in MEGA 5.10. For ML, 1,000 bootstraps and all sites of the alignment were used with a Kimura-2-parameter (K2P+G+I) model (found as the best model in MEGA 5.10 with lowest BIC) corrected with gamma distributed rates among sites (with 10 categories from 0.0001 to 5.8431) and a proportion of invariant sites of 0.1184, as deducted from the dataset (shape parameter 0.2504). Trees were inferred by extensive Subtree-Pruning-Regrafting (SPR) with an automatic initial NJ tree and strong branch swap filter. MP was run using all sites and 1,000 bootstraps with SPR tree searching (10 initial trees, search level 1). NJ was used with the K2P model, uniform rates among sites and the pairwise deletion option for 1,000 bootstrap replicates to obtain a consensus tree. Bayesian Phylogenetic Inference was carried out using Mr.Bayes 3.2 [29] with default settings under a GTR+G+I model with 10 gamma rate categories for 2,000,000 generations, with a sample, print and diagnostics frequency of 500, 500 and 1,000 respectively and a relative burnin of 25%. A topology prior was set for the ingroup (all but *Emiliania huxleyi*) as a hard constraint. For *rbc*L marker analysis, the set of taxa from [11] was used. Taxa were specifically chosen such that for each taxon the same isolate was used for *rbc*L and 18S analyses (see S1 Table). Sequences from this study were added by clustalW alignment under codon translation, as implemented in BioEdit 7.0.5.3 [30]. The whole *rbc*L alignment contained 1231 bp with 9.68% missing data and only one gap of 36 bp in AF438319. ML analysis was carried out using raxmlGUI 1.3 (with RaXML7.4.2, [31,32]) with data partitioned into the 3 codon positions with per partition branch length optimization for 10 runs with 100 thorough bootstraps each and the GTRGAMMAI model. NJ was calculated using the K2P model, uniform rates among sites and pairwise deletion option under 1,000 bootstrap replicates. For MP analysis all sites were used under SPR searching (10 initial trees, search level 1). Both NJ and MP were calculated with MEGA 5.10. Bayesian Phylogenetic Inference was carried out using MrBayes 3.2 with default settings and 10 gamma rate categories under the GTR+G+I model, also with data partitioned into 3 codon positions and unlinked rates of reversible rate matrix, stationary state frequencies, gamma distribution shape parameter and proportion of invariable sites. 10,000,000 generations were run with a sample, print, and diagnostics frequency of 500, 500 and 1,000 respectively and with a relative burnin of 25%. All *rbc*L calculations were repeated under removal of either *C*. *socialis* or *G*. *wildpretii*. Saturation plots of 1^st^, 2^nd^ and 3^rd^ *rbc*L codon positions only were created in R by plotting pairwise GTR+G+I corrected distances against p-distances, as calculated in Mega 5.10 and raxmlGUI 1.3 (with RaXML7.4.2) under pairwise-deletion.

To account for compositional bias in the 18S rDNA dataset (as seen from disparity index results and GC-content comparisons), 3 approaches were used: RY-coding, LogDet transformation and ML under a nonhomogeneous non-stationary substitution model (see supplementary methods S1 Text). For all 18S rDNA and *rbc*L tree construction methods, *E*. *huxleyi* was used as a root.

The molecular clock hypothesis in the final 18S dataset was evaluated independently of the time dimension with Tajima’s relative rate test [33]. Sequence comparisons of all used Chryso-/Synuro- and Synchromophyceae were carried out using R 3.0.0. with additional packages ape 3.0–9 and pegas 0.5 [34]. P-values were corrected for multiple comparisons using the Holm-Bonferroni method [35] implemented in the stats package. Bayesian Phylogenetic Inference of the final 18S dataset was then repeated as described above, implementing relaxed clock assumptions. The model was set to GTR+G+I with 10 gamma categories. A topology prior was set for the ingroup (all but *E*. *huxleyi*) as a hard constraint. We specified an underlying uniform clock model with the IGR (independent gamma rates) model for variation of rates across lineages. 6,000,000 generations were run with a sample, print and diagnostics frequency of 500, 500 and 1,000 respectively and a relative burnin of 25%.

### Nomenclature

The electronic version of this article in Portable Document Format (PDF) in a work with an ISSN or ISBN will represent a published work according to the International Code of Nomenclature for algae, fungi, and plants [36], and hence the new names contained in the electronic publication of a PLOS ONE article are effectively published under that Code from the electronic edition alone, so there is no longer any need to provide printed copies. The online version of this work is archived and available from the following digital repositories: PubMed Central, LOCKSS.

## Results

### Taxonomic treatment

*Guanchochroma* Schnetter et Schmidt gen.nov: Unicellular, amoeboid alga with chlorophylls a and c. Number of plastids increased with age of the cell. Main cell body (MCB) with all chloroplasts and nucleus encased in hyaline lorica. Colony (meroplasmodium) formation by fusion of reticulopodia, all protruding through ostioles of loricae. Initial size of lorica expanding by secondary growth. Increase in thickness by basting of new lorica to inner surface of existing lorica. Vegetative reproduction by binary division of MCB inside loricae and hatching of one or both daughter cells through an ostiole as migrating amoebae without flagellae. Ostiole sealed by additional lorica produced by remaining daughter cell. Cells with secondary, multilayered walls are main dispersal units (cyst-like stages). Thylakoids in plastids arranged in loose stacks of three. Girdle lamellae and pronounced pyrenoids missing.

Type species: *Guanchochroma wildpretii* Schnetter et Schmidt

Etymology: The genus name *Guanchochroma* is a reference to the Island of Tenerife where the type locality is situated [Guanche(s): name given to the aboriginal people of Tenerife], and its pigmentation with chlorophylls a and c (chroma: Greek noun = color).

*G*. *wildpretii* Schnetter et Schmidt spec. nov.: MCB surrounded by globular, stem-less lorica with usually one short-rimmed ostiole. Sessile cells with initially 6–10 plastids and lorica diameter of 10–12 μm, increasing in size up to usually 25 μm. Hatched (migrating) amoebae moving along or close to branches of reticulopodial network. Settling of amoebae and formation of new, single-layered loricae close to existing network, followed by re-attachment to it. Floating amoebae and gametes not observed. Formation of small accumulations of densely grouped cells under culture conditions.

Type: Type material was deposited at the Herbario La Laguna (TFC, Universidad de La Laguna, Tenerife, Spain) as TFC Phyc. 14906A (holotype, block of plastic embedded cells) and 14906B-E (isotypes, semithin sections on object slides). Distinctive morphological features of *G*. *wildpretii* illustrated in Fig 1.

Type locality: Type material was collected by R. Schnetter from a basaltic rock tide pool in the upper sublittoral, between the light house of Punta de Hidalgo and the Roca Negra (Tenerife, Canary Islands, Spain) in November 2009 (latitude 28.57248°, longitude -16.33211°). R. Schnetter obtained the unialgal isolate from this material.

Etymology: The epithet wildpretii was chosen in honor of Prof. Dr. Dr. h. c. Wolfredo Wildpret de la Torre (* September 16, 1933) who established the Departamento de Biología (Botánica), with marine botany as one of its fields of research, worked meritoriously as dean of the Facultad de Biología and director of the Centro Superior de Ciencias Agrarias at the Universidad de La Laguna (Tenerife), and was tirelessly engaged in projects for the conservation of natural environments of the Canary Islands.

### Morphology of *G*. *wildpretii* compared to *C*. *socialis*

#### General morphology

**Fig 2:**
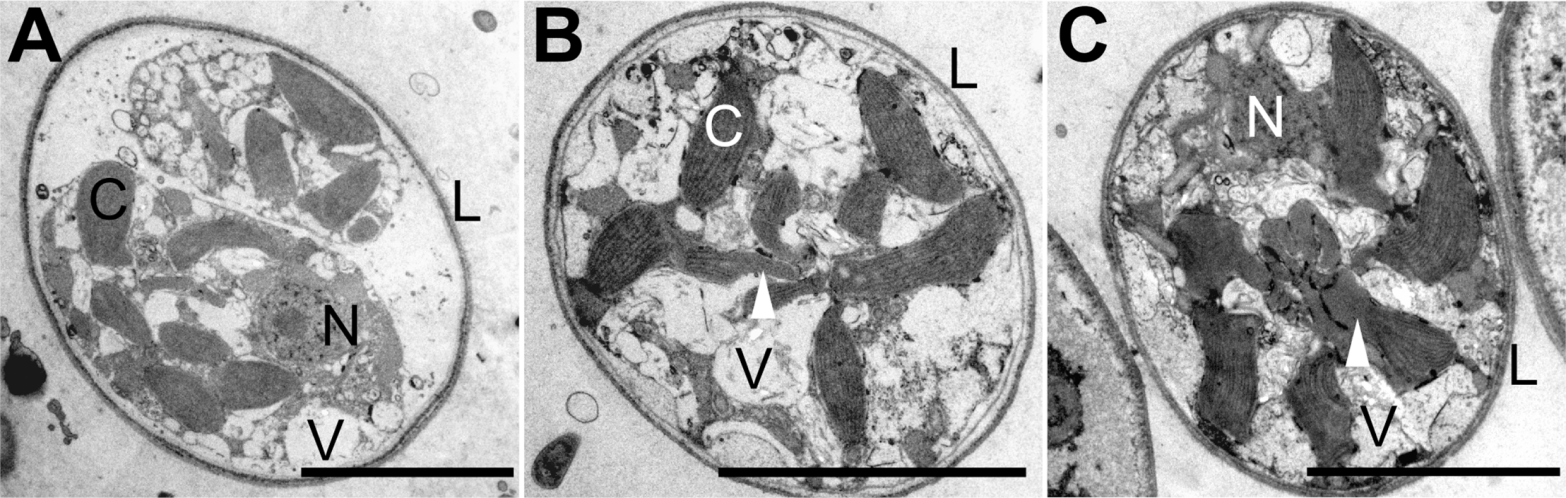
Arrangement of plastids in *Guanchochroma wildpretii*. (A) TEM of two amoebae in one multilayered lorica after binary division. The nucleolus is distinct within the nucleus. Vacuoles, mostly roundish, are present in different sizes. The plastids show no pyrenoids and are not interconnected. (B) TEM of an amoeba within a multilayered lorica. Vacuoles are present, yet with highly variable outline. Some plastids show terminal elongations without thylakoids (arrowhead). No Nucleus is visible in this section plane. (C) TEM of an amoeba within a multilayered lorica, showing four plastids and a nucleus without nucleolus in the section plane. Plastids possess tubular to globular shaped protrusions (white arrowhead) that group in the cell center (black arrowhead). The cytoplasm appears highly granular with variably shaped, non-globular vesicles. V—vesicle, N—nucleus, L—lorica, C chloroplast. Scale bars 5 μm.

*Guanchochroma wildpretii* was identified as a unicellular, amoeboid eukaryote with a close resemblance to *Chrysopodocystis socialis*. Most cells of *G*. *wildpretii* are sessile amoebae, whose MCBs are encased in globular, hyaline loricae. The reticulopodium of the cell can exit the lorica via usually one ostiole with a short rim, which is located laterally, pointing slightly towards the surface (Fig 1A, 1H and 1I). Reticulopodia of different cells may fuse, creating a so called meroplasmodium, in which particles such as food vacuoles are transported to the MCBs (see Fig 1D and 1E for an overview and close-up of a meroplasmodium). Asexual reproduction of cells occurs by binary division inside the lorica (Fig 2A), after which one cell leaves the lorica of the mother cell as a migrating amoeba by hatching through the ostiole. Optionally, the second amoeba can also hatch. Migrating amoebae of *G*. *wildpretii* can take various shapes depending on the direction and speed of their movement. The shape ranges from almost spherical cells with various axopodia-like filopodia to fusiform cells, with a leading and a trailing filopodium. This description is similar to the conditions found in *C*. *socialis* and, apart from the lorica shape, also for the *Synchroma* species.

#### Plastids

*G*. *wildpretii* initially possesses 6–10 plastids within each MCB with apparent non-random distribution. After the settlement of migrating amoebae and the excretion of the lorica, the plastids form groups close to the center of the cell creating a star-shaped three-dimensional arrangement with plastid lobes pointing outward and vacuoles adpressed to the cell membrane between these lobes (Fig 1E). The plastids are lenticular, sometimes with irregular, curved outlines and are about 2.5 μm wide and 3.5 μm long. Older cells are packed with >10 plastids and their arrangement is less clear due to the presence of multiple vesicles directly below the cell membrane (see below, Fig 1F). In some section planes of sessile cells with multilayered loricae, an aggregation of plastids is visible (Fig 2B and 2C). Non-granulated, elongated protrusions of plastids are situated closely to the cell center. TEM analyses show that the membrane conformation of these structures does not comply with the definition of the plastid complex, while plastids are surrounded by 4 membranes each (S7 and S8 Figs). Since clear evidence for a plastid complex membrane structure is missing, especially in young cells, and a central pyrenoid group is absent, it will be referred to as a plastid group. The pigmentation detected for *G*. *wildpretii* was in accord with pigments found in heterokont algae and included chlorophylls *a* and *c*_1+2_, fucoxanthin, violaxanthin, antheraxanthin, zeaxanthin and beta-carotene (S11 Fig).

**Fig 3:**
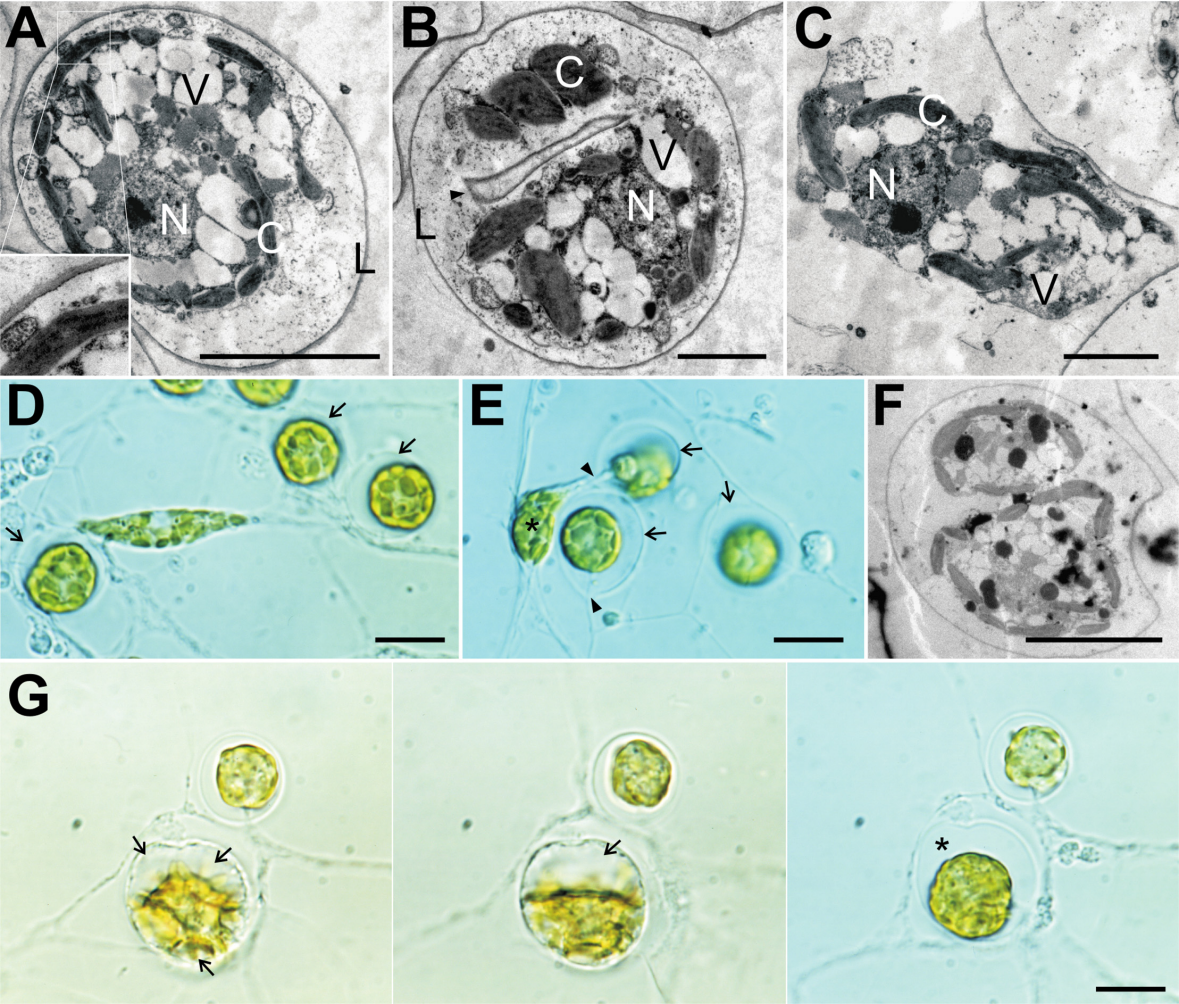
Morphology of *Chrysopodocystis socialis*. (A) TEM of sessile amoeba encased in a thin lorica. Central nucleus surrounded by multiple vacuoles. Parietal chloroplasts are pressed against the cell membrane. (B) TEM of one amoeba in a lorica during cytokinesis with a diaphragm-like structure (arrowhead). (C) TEM of migrating amoeba moving between two loricae. Chloroplasts are flattened against the cell membrane. (D+E) Migrating amoeba with leading and trailing filopodium. Chloroplasts are randomly distributed in the cell and the nucleus positioned in the center. Sessile amoebae encased in globular, hyaline loricae (arrows). After binary division, one daughter cell (asterisk) leaves the lorica through the ostiole (arrowhead). In some focus planes, the short rim of the ostiole is visible. (F) TEM of two sessile amoebae in one lorica after binary division. (G) Sequence of lorica formation from establishment of multiple vacuoles (left, 9:55 PM), the fusion into one vacuole (center, 10:30 PM), to the complete reduction of vacuoles and the re-establishment of a connection to the meroplasmodium (right, next day 4:45 PM). The final lorica is considerably larger than the MCB, leaving room (asterisk) for expansion during cell growth. Scale bars A, F: 5 μm, B, C: 2 μm, D, E, G: 10 μm. C—chloroplast, N—nucleus, L—lorica, V—vesicle.

The cells of *C*. *socialis* were also investigated for structures similar to the chloroplast complex found in the *Synchroma* species. EM analysis of ultrathin sections confirm observations made using light microscopy: the lenticular plastids (about 0.5 μm wide, 2.5 μm long) are separately distributed in the cells and usually adpressed longitudinally to the cell membrane due to the presence of multiple vacuoles close to the cell center (panel H in S10 Fig). No connections between plastids were visible and each cell possessed about 20 plastids without apparent pyrenoids (Fig 3A; S10 Fig).

#### Lorica

In addition to plastid distribution, the main difference between *G*. *wildpretii* and *C*. *socialis* concerning general morphology was found in the behavior of migrating amoebae and the appearance of the lorica. The migrating amoebae of *C*. *socialis* (Fig 3D and 3E) move solitarily and independently of the meroplasmodium and frequently detach from the surface to form floating amoebae (heliozoan-like cells). Together, migrating and floating amoebae constitute the main mode of dispersal of this species. Migrating amoebae of *G*. *wildpretii* usually stay close to the existing meroplasmodium, maintaining a physical connection via their filopodia (Fig 1C) or moving entirely on top or even in the existing meroplasmodium (Fig 1B) and are therefore prone to settle close to the meroplasmodium. Floating amoebae, i.e. naked amoebae detaching from the surface, were not observed in *Guanchochroma*. Hence, *C*. *socialis* has the potential to found new colonies with a considerable distance to mother colonies, while the migrating amoebae of *G*. *wildpretii* only contribute to the radial growth of their mother colonies.

Sessile cells of *C*. *socialis* show a succession of events during and after lorica excretion similar to the *Synchroma* species. During settlement, the migrating amoeba rounds off, takes up water into multiple large vacuoles (thereby increasing the MCB size) and excretes the globular lorica. Afterwards, these vacuoles fuse to form a large one, which is successively reduced in size (Fig 3G). These events lead to a lorica (10.9 ± 0.8 μm, n = 100) which is considerably larger than the containing MCB, and leave a distinct water-filled space between lorica and MCB (Fig 3F; panels A1 and I in S10 Fig). The lorica is made up of an electron dense inner and a loosely packed outer part, yet it is at all times only single-layered (Fig 3A; S10 Fig).

Settling amoebae of *G*. *wildpretii* take on a globular shape, retract their filopodia completely and increase their volume by taking up water in multiple, parietal vacuoles (Fig 1E) as well. After the excretion of the lorica, the MCB volume may decrease again by the reformation of the reticulopodium, but a pronounced water-filled space between the cell membrane and the inner surface of the lorica is not visible, except for the one found directly in front of the ostiole (Fig 1A and 1I, sequence of settlement Fig 1C). The initially excreted lorica has a diameter of 10.8 ± 1.2 μm (n = 100). Despite settlement differences, the initial lorica sizes of *C*. *socialis* and *G*. *wildpretii* are not significantly different.

*G*. *wildpretii* readily forms sessile cells with thick loricae and highly granular MCBs. These cells are larger than freshly settled cells (Fig 1D) and indicate the presence of a lorica expansion mechanism. During this secondary growth beyond the initial lorica size the number of chloroplasts per cell also increases and multiple vesicular compartments at the cell periphery are present, probably containing new lorica material for expansion. An ostiole is not always visible and transfers of these cells to new culture medium result in the hatching of multiple migrating amoebae and formation of a new colony. Besides their expansion, the loricae in *G*. *wildpretii* can be multilayered as well (Fig 1H and 1I; S6 Fig). After binary division and hatching of one migrating amoeba, the remaining amoeba excretes a new lorica within the old one (panel F in S6 Fig). Through its subsequent expansion, the new lorica will adhere to the inner surface of the old one, adding a layer to it. Apparently, the ostiole can be sealed during the addition of layers and a new one formed (even at another position), which can be deducted from some TEM section planes (Fig 1H and 1I; panel K in S6 Fig). In lab cultures the diameter of secondary loricae reaches a maximum at approximately 25 μm, i.e. equivalent to about 2.5 times the size of the initial lorica. EM data reveal multiple layers with different electron densities making up these secondary loricae (Fig 1I; S6 Fig). While loricae of both species are usually firmly affixed to the substrate surface, multilayered cell stages of *G*. *wildpretii* can detach from the surface more readily and float in the medium. By re-attaching to the surface at a different site they serve as the main means of long-range dispersal. In old cultures, contents of cells without rhizopodia can turn dark and act as resting (cyst-like) stages. After a transfer into new medium they may form rhizopodia again.

### Marker analysis

#### Marker length and composition

The alignment of an 18S rDNA heterokont dataset obtained by adding sequences of *C*. *socialis* and *G*. *wildpretii* to a structural alignment from the SILVA database revealed the presence of multiple insertions for these two species, which could be pinpointed to variable loop regions of the 18S secondary structure model available for the heterokont class Bacillariophyceae. The two larger insertions were found in variable regions V2 (5’ end) and V8 (3’ end) (see S1 Fig). The position of a 3’ insertion was shared between *C*. *socialis* and *G*. *wildpretii*, while the 5’ insertion was unique to *C*. *socialis*. The resulting secondary structure of region V8 was modelled for Synchromophyceae and related amoebae using mfold (see S2 Fig). The general positioning of helices 45 and 46 that make up region V8 was similar for the analyzed amoeboid species, except for *G*. *wildpretii* and *C*. *socialis*. Their helix E46 position was shifted due to an insert (designated helix E45-1 between helices E45 and E46) of 42 and 50 nt, respectively. While the presence of this insert was common among *Guanchochroma* and *Chrysopodocystis*, homolog positions could not be identified with certainty because a secondary structure reference is missing. Furthermore, paired bases of helix 46 showed a shared unique sequence (CUGCgg(c/a)aGCAG, paired bases in capitals), explicitly different from all closely related species and even all other available eukaryotic datasets.

The nucleotide composition of 18S rDNA sequences of *C*. *socialis* and *G*. *wildpretii* was assessed by comparing the GC-content of all eukaryotes for which homologous sequences were available from the SILVA database in the positional range of Synchromophyceae. The overall GC-content of eukaryote 18S rDNA in the chosen segment ranged from 27.5 to 66% and their corresponding lengths from about 1100 to 3250 nt (see S3 Fig). With 1708 and 1639 nt in length and 52.7 and 53.9% in GC-content for *C*. *socialis* and *G*. *wildpretii*, respectively, these species lie well within the wide eukaryote range. Yet in comparison to the subset of stramenopiles sequences they represent the maxima for GC-content reported thus far. Inspection of *rbc*L length and composition within the dataset revealed no bias as observed for the 18S rDNA dataset.

Since length and GC-content of the 18S gene suggested an altered substitution pattern for both species, the disparity index test (test for homogeneity of substitution pattern) was used to systematically identify different underlying mechanisms of evolution for both of the used markers (*rbc*L and 18S rDNA) of the analyzed species. For the 18S dataset significant differences were detected between *C*. *socialis* and all other taxa, as well as for *G*. *wildpretii* compared to all other taxa (S2 Table). Yet, between these two species no difference in substitution pattern was found, indicating that they are more similar to each other than to all other taxa concerning the evolution of their 18S rRNA gene (see S2 Table). Highest disparity indices (>4.0) and therefore large substitution pattern differences were found between *C*. *socialis*/*G*. *wildpretii* and the Bolidophyceae, Chryso-/Synurophyceae and Oomycetes. For disparity index tests of the *rbc*L dataset mean indices per site were always < 2.0, mostly even < 1.0, and the corresponding tests showed significant substitution heterogeneity for about 58% of the species compared to *C*. *socialis* and 41% of the species compared to *G*. *wildpretii* (S2 Table). However, the differences were not restricted to certain taxa, albeit they were mostly found in comparisons to the Chryso-/Synurophyceae.

Overall, the 18S sequences of *C*. *socialis* and *G*. *wildpretii* were marked by a common high GC-content, shared insert positions, an identical helix 46 and a similar substitution pattern.

#### 18S and rbcL phylogeny

**Fig 4:**
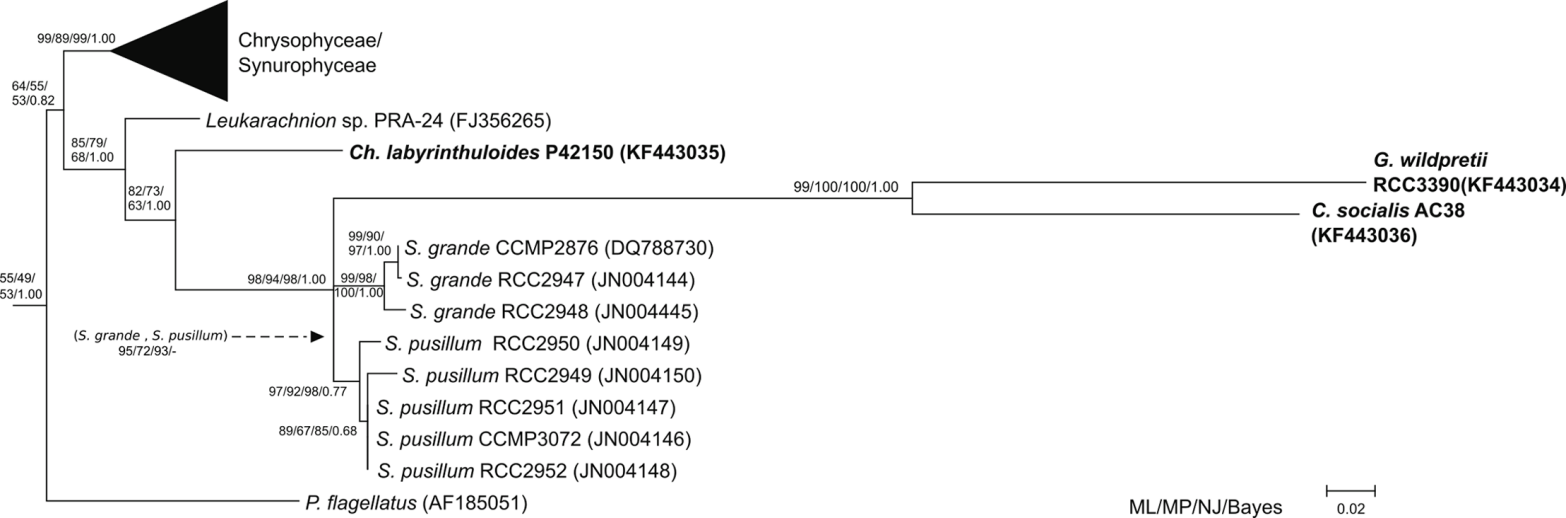
Phylogenetic tree of stramenopiles using 18S rDNA data to position *Guanchochroma wildpretii* and *Chrysopodocystis socialis*, Based on 78 heterokont taxa with 1506 positions and rooted with *E*. *huxleyi*. A Maximum Likelihood subtree with Synchromophyceae and related organisms is shown. Support values (bootstrap, posterior probabilities) given at nodes for all used methods. ML—Maximum Likelihood, MP—Maximum Parsimony, NJ—Neighbor Joining (K2P model), Bayes- Bayesian Analysis. Names of taxa given with corresponding accession numbers. Sequences from this study highlighted in bold, all other sequences according to dataset used by [11].

To elucidate the position of *C*. *socialis* and *G*. *wildpretii* among other heterokont algae, the nuclear (18S rDNA) and plastid (*rbc*L) markers were used for phylogenetic inference. The 18S rRNA gene alignment comprised 78 taxa and 1506 positions after being treated with gblocks to remove ambiguously aligned and substantial indel positions (see Methods). For all general inference methods used (ML, MP, NJ, Bayes), we recovered an *S*. *grande* and an *S*. *pusillum* clade, both with moderate to high support values, as well as a clade comprised of *C*. *socialis* and *G*. *wildpretii* (*CG* clade) with high support. While these three clades themselves were thus well differentiated, we found the support for a *Synchroma* clade to decrease with the addition of *G*. *wildpretii* and *C*. *socialis* to the dataset (S3 Table). Additionally, its branch was very short, leading to a polytomy within the *Synchroma* + *Chrysopodocystis* + *Guanchochroma* (*SCG*) clade for Bayesian analysis (Fig 4). Independently of the used subset (excluding either *G*. *wildpretii* or *C*. *socialis*) the same topology was obtained, except for Bayesian analysis, where *G*. *wildpretii* and *C*. *socialis* then fell within and not as a split off to the *Synchroma* clade. The supports for the *SCG* clade were high under all methods used (see S3 Table for respective support values in reduced datasets). As seen in previous 18S rDNA studies, *Ch*. *labyrinthuloides* remained a sister clade to Synchromophyceae and therefore also to the *SCG* clade within our data set, while *Leukarachnion sp*. PRA-24 was consistently recovered branching off before the above taxa. The supports for this topology were moderate in all but Bayesian Analysis, where it received maximum support.

To exclude the effect of similar base composition (parallel compositional heterogeneity) as a source of *G*. *wildpretii* and *C*. *socialis* clustering in the previous calculations, different approaches were used (see S1 Text). Overall, none of the additional methods for 18S inferences was able to improve support for the branching order within the *SCG* clade, yet, the previously found topology persisted. Also, Bayesian analysis using a relaxed clock model for the 18S rDNA dataset (S6 Fig) did not resolve the trifurcation of the *SCG* clade for this method with certainty. Yet, the analysis showed that rates along branches were increased for terminal branches to *G*. *wildpretii* and *C*. *socialis*. Rates for internal branches were also highly increased for the *CG* and the *SCG* clade.

**Fig 5:**
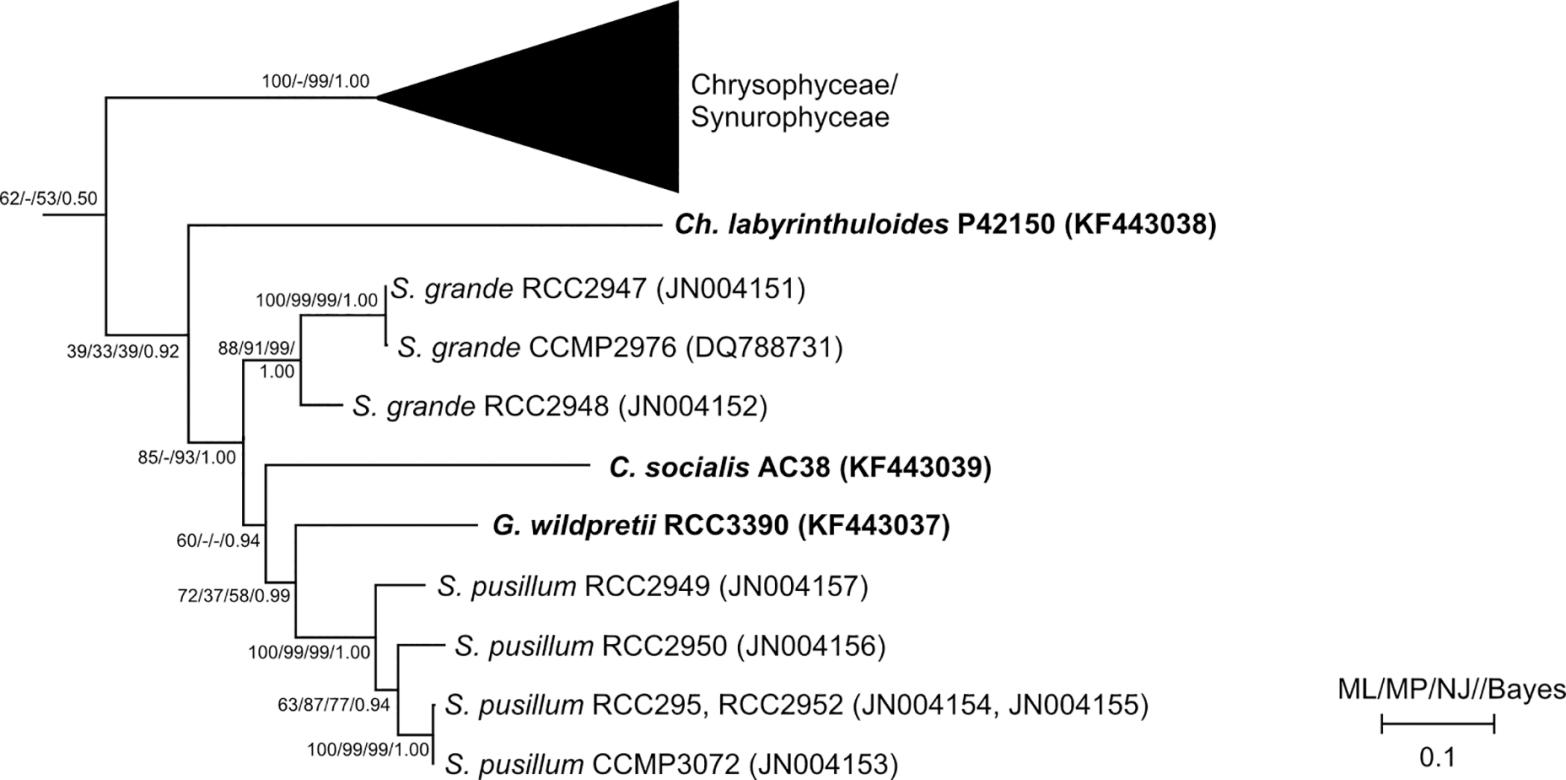
Phylogenetic tree of Ochrophyta using *rbc*L data to position *Guanchochroma wildpretii* and *Chrysopodocystis socialis*. Based on 74 photosynthetic taxa with 1231 positions. Maximum Likelihood subtree of Synchromophyceae and related photosynthetic organisms, with ML, MP and NJ bootstrap values and Bayesian posterior probabilities given at nodes. Formatting as in Fig 4.

In contrast, the plastid encoded *rbc*L gene did not show deviations in GC-content, length or substitution pattern (S2 Table) in comparison to the remaining heterokont dataset. However, due to sequence conservation and limited sequence length, the *rbc*L dataset did not improve the resolution within the *SCG* clade. Two main differences to the 18S dataset became apparent. The *Guanchochroma* and *Chrysopodocystis* species did not form a robust clade, i.e. indicated no immediate common ancestor based on the *rbc*L marker. Instead, *G*. *wildpretii* diverged before the *S*. *pusillum* clade and *C*. *socialis* branched off before that grouping (Fig 5). The branch lengths of these splits, however, were short and support values (bootstrap and posterior probabilities) generally low. Saturation plots of p-distances against corrected distances for each codon position showed that the 3^rd^ codon position had overall the highest saturation, yet the curvature was not strong enough to suggest an excess of non-phylogenetic signal. The addition of *Ch*. *labyrinthuloides rbc*L data did not improve the resolution of splits. However, it confirmed the affinity of this species with Synchromophyceae. Branching of *Ch*. *labyrinthuloides* was similar to that in 18S rDNA results (Figs 4 and 5).

In summary, the phylogenetic branching order of *G*. *wildpretii* and *C*. *socialis* in relation to the *Synchroma* clade based on two molecular markers was in agreement with morphological findings for the nuclear marker and unresolved for the plastid marker.

## Discussion

Through morphological analyses of a new amoeboid algal isolate from Tenerife, we found that this unicellular organism has a unique combination of characteristics and qualifies as a new species and genus–*Guanchochroma wildpretii*. A close relationship to *Chrysopodocystis socialis* was suspected due to shared features such as initial lorica size and globular lorica shape, formation of the meroplasmodium and binary cell division inside the lorica. Differences only became apparent in long-term observations, where *Guanchochroma* forms cyst-like cell stages which act as the main dispersal units instead of migrating and floating amoebae, which were shown to be typical for *Synchroma* and *Chrysopodocystis* [12,14]. The cyst-like stages possess multilayered loricae and show a secondary growth in which the lorica expands in unison with MCB growth. While the addition of layers to the inner surface of loricae has been shown previously for example in the chlorarachniophyte species *Lotharella vacuolata* [37], *L*. *globosa* [38] and *L*. *reticulosa* [39], the growth of loricae has not. This expansion is unique for loricae, which are usually more or less opened to one side, retain a constant size after formation and are found in distantly related eukaryotic groups such as *Dinobryon* (Ochrophyta) [40], *Strombomonas* (Euglenophyta) [41] and choanoflagellates [42]). Whether loricae of *G*. *wildpretii* retain one or more ostiole(s) at all times is not known. Yet these walled cells function as long-range, long-term dispersal units. A prolonged closing of ostioles during this process seems likely due to increased resistance to osmotic variability frequently encountered in the habitat of this species, the coastal tide pools. The chloroplast arrangement also serves as a distinguishing feature. While neither of the two species possess a plastid complex, approximately 20 chloroplasts are randomly distributed in *C*. *socialis* at all times, while initially 6–10 plastids are grouped in the cell center for freshly settled sessile amoebae in *G*. *wildpretii*. Even though this plastid group is superficially reminiscent of the plastid complex found in Synchromophyceae, it does not possess the required membrane topology. Yet both chloroplast arrangements could aid in the coordination of synchronized plastid division.

Apart from morphological similarities between *C*. *socialis* and *G*. *wildpretii*, they also share insertions at several positions in their 18S gene which increase the length of the gene in the studied region by about 200 and 150 nt, respectively, and neither their sequence nor assumed secondary structure appears similar. The four most pronounced insertions in the complete eukaryotic 18S rDNA dataset are spread among four different supergroups (*Gyropaigne*, Excavata; *Goniomonas*, CCTH clade/Cryptophyceae; *Acanthamoeba*, Amoebozoa; *Ammonia*, SAR/Rhizaria). While elongations of the 18S gene at this position are frequently found among all eukaryotic supergroups, their functions are seldom discussed in detail [43,44] (but see [45]). The general presence of inserts specifically at this position must therefore be considered paralogous, while the reasons and functions for these expansions remain unclarified. Yet the fact that they can be found in the organisms presented here (with many similar morphological features), together with the shared, unique sequence identity of the adjacent helix 46, make a common descent of *G*. *wildpretii* and *C*. *socialis* likely when judged by these features alone.

In addition the abovementioned 18S gene elongation in *C*. *socialis* and *G*. *wildpretii*, this gene also showed an increased GC-content. This feature is not caused solely by GC-biased insertions. A genome-wide, punctual increase in GC-content is speculated to be caused by biased gene-conversion (BGC) and not necessarily selection processes (be they positive or negative) [46], and in humans BGC has been associated with specific loci that show an increase in recombination rate [47]. For the rRNA operon, this increased recombination rate is achieved by concerted evolution, i.e. the gene conversion between paralogs [48]. Escobar et al. [49] showed that, over a broad spectrum of eukaryotes, an increase in rRNA GC-content is indicative for a genome wide GC biased gene conversion (gBGC). To what extent the observed increased GC-content in the *CG* lineage can also be assigned to other loci or even the whole genome remains to be seen by sampling protein-coding, well-studied genes such as those for beta-tubulin, actin or hsp90. From studying the *rbc*L gene in whole or any of its codon positions alone, it seems clear that the plastome is not affected by BGC. In total, it remains unclear to which evolutionary processes the 18S elongation and GC-content increase could be attributed.

The mostly limnic Chrysophyceae formerly comprised also the marine species *C*. *socialis*. Through plastid and nuclear marker analysis, and also morphology, we showed that it is more closely related to the Synchromophyceae than to the Chrysophyceae, even though a plastid complex is missing. The same placement could be found for the new species *G*. *wildpretii*, which represents a sister clade to *C*. *socialis*, highly supported by 18S molecular and morphological data. Long branches on the 18S rDNA tree and shared GC bias for these species did not attribute to this placement, as all methods used to counteract these effects recovered this topology with high to maximum support. It is generally accepted that an increase in substitution rate along specific branches of phylogenetic trees as observed for the *CG* clade, can be attributed to one or multiple factors. Exemplary contributing factors are the change in functional constraints of proteins/RNA [50], metabolic rate [51], temperature [52], sociality [53], generation time and population and genome size [54]. For *Chrysopodocystis* and *Guanchochroma* population size could have contributed to the observed substitution rate increase. While Koch et al. noted that the benthic lifestyle of these plasmodial algae must increase the chance of cell-cell encounters with possible exchange of genetic material through fusion of haploid or diploid amoebae [12], it also increases the chance for allopatric speciation events. This occurs through the drift of dispersal units (cysts or floating amoebae) or a small cell colony on marine debris to a previously unoccupied tide pool and the establishment of a new population from only few, probably clonal cells. Without contact to other colonies due to restricted sea water mixture between pools or reduced dispersal cell type formation, speciation can take place. Such a founder effect represents one possible explanation for the fixation of increased substitution rates in the *CG* clade, due to genetic drift acting upon a reduced effective population size.

The *rbc*L marker is often used in addition to 18S rDNA and has helped in the detection of secondary plastid origins, for example in the Chlorarachniophyte lineage [55]. The results of our phylogenetic calculations obtained through the plastid encoded marker *rbc*L did not attain the support values found for the nuclear one. Reduced phylogenetic resolution of *rbc*L versus 18S rDNA data for a comprehensive Chrysophyceae dataset has been reported previously [56] and there the observed saturation in 3^rd^ and 1^st^ codon positions was thought to result from positive selection of the *rbc*L gene during the radiation of Chromist lineages. Yet our saturation plots did not show strong curvature which would indicate problematic fast-evolving sites for phylogenetic analysis in the present *rbc*L dataset. Additionally, preliminary phylogenetic analyses of further plastid genes *psb*D and 16S rDNA indicate similar deviations from 18S analysis and display low support values for splits within the *SCG* clade (data not shown). The found low phylogenetic resolution can be attributed to an insufficient number of phylogenetically informative sites and explained by the rapidly occurring, successive speciation events found within the *SCG* clade as indicated by the 18S analyses, coupled with a reduced substitution rate in organelles compared to the nucleus. In general, the substitution rate of organelles e. g. in higher plants is considerably lower than the nuclear one [57]. While for other photosynthetic eukaryotic groups a broad range of absolute and relative rates can be found [58,59] (see also [54]), so far only organelle but not nuclear loci were inspected for rate differences in heterokont algae [60]. Low substitution rates in highly conserved protein-coding genes and mutational saturation have led to the sequencing of larger and also non-coding genetic regions in more comprehensive studies [61]. Nevertheless, the plastid gene supports the ancestry of *G*. *wildpretii*, *C*. *socialis* and the *Synchroma* species found for the nucleus encoded 18S rRNA gene.

With limited resolution of *rbc*L data, the position of *Guanchochroma* and *Chrysopodocystis* in relation to *Synchroma* must be deduced from morphology and 18S data. We consistently found a *Synchroma* clade (95/72/93 for ML/MP/NJ respectively) to the exclusion of *Guanchochroma* and *Chrysopodocystis*. This topology also seems most likely, given the tremendous morphological similarity of *S*. *grande* and *S*. *pusillum*. The verification of this positioning can be achieved by the sampling of further amoeboid organisms from this group. One example could be the genus *Reticulosphaera* [62–64] with remarkable morphological similarity to *Synchroma*. Unfortunately, TEM data, sequence records and, to our knowledge, type material in culture collections are no longer available.

Since neither *C*. *socialis* nor *G*. *wildpretii* possess the key feature of the Synchromophyceae, they do not comply with the class definition or subsequent ranks [9] and demonstrate difficulties arising from arbitrarily chosen taxonomic rank systems where, in fact, no clean-cut groups, but only gradual change exists. However, a close ancestry is certain, even based on morphology alone and the analysis of 18S sequence data revealed that the speciation of *Synchroma* and the *CG* clade split-off, happened in close temporal proximity. As an external lineage to the *Synchroma* species, two evolutionary scenarios are conceivable. The first and most parsimonious one states that they never had the plastid complex. The second involves the secondary loss of this unique class feature. Our preferred hypothesis of evolutionary events comprises the split of the *CG* clade before the chloroplast complex occurrence in *Synchroma*, while *Chlamydomyxa* and *Leukarachnion* split off even earlier. Yet, a secondary loss of plastid complexes in the *CG* lineage has to be considered as well, especially since chloroplasts are grouped in settling amoebae of *G*. *wildpretii*. Information on division of secondary plastids in the heterokont lineage is scarce and focused on morphological stages [65,66]. While data on chloroplast-targeted proteins involved in the fission of the outer membrane pair is not available to date [67], possible candidates include proteins generally involved in eukaryotic plasma membrane and vesicle fission, e.g. dynamins, protein kinase D, small G proteins among others. Since all plastid membranes in Synchroma do divide eventually and two daughter complexes are formed before cytokinesis, a mere loss-of-function mutation in a division machinery component seems unlikely for complex establishment. As a consequence, a regulatory feature must be the key aspect and a reversion to the original state (single plastids each with its own inner three membranes and randomly distributed within the MCB) is hypothetically possible.

Further investigation of the plastid division machinery or comparisons of plastomes, more nuclear data or whole genomes of the closely related taxa presented here may reinforce the position of *G*. *wildpretii* and *C*. *socialis* in relation to *Synchroma* and settle the question of the *CG*-clade including derived Synchromophyceae (loss of plastid complex) or representing a basal branch to the Synchromophyceae. Until then we refer to *G*. *wildpretii* and *C*. *socialis* as Synchromophyceae-related organisms and leave their higher order classification open to further evidence and investigation. As stated by Koch et al. [12], the Synchromophyceae are equipped for rapid diversification in the benthic habitat. This class and close relatives indeed show a highly diverse morphology, ranging from purely amoeboid and obligate heterotrophs (*Leukarachnion*), over multiple plastid-bearing, limnic amoebae (*Chlamydomyxa*), to mostly sessile, plastid grouping organisms (*Synchroma*). Their ancestry has been shown before and although supports for some splits decreased with the addition of highly divergent *Chrysopodocystis* and *Guanchochroma* 18S rDNA data compared to previous studies [8,10,11], the general topology persisted. All abovementioned genera are united by the common presence of the meroplasmodium, loricate amoeboid cells and typical heterokont pigmentation when plastids are present, while flagellar cell stages are missing (see Table 1).

## Conclusion

We present a new amoeboid, photosynthetic species, *Guanchochroma wildpretii*, and also the former chrysophyte *Chrysopodocystis socialis*, both with strong affiliation to the heterokont algal class Synchromophyceae (Ochrophyta). The used phylogenetic markers showed a robust grouping of *Guanchochroma* and *Chrysopodocystis* with *Synchroma*, albeit without fully resolving the order of splits for the plastid marker due to rapid divergence within this group. Since both species possess individual plastids not grouped into complexes inside a common outer membrane pair, they represent two additional genera for the further study on plastid complex establishment, by comparison of potential plastid division machinery components with the two *Synchroma* species. Other scientific questions could include the study of biased gene conversion in eukaryotes with secondary plastids, substitution rate heterogeneity within and between genomes of eukaryotes with secondary plastids and the rapid adaptation and speciation processes observed for benthic algae. It remains to be seen if other algae can be associated with the Synchromophyceae and if they bear a plastid complex. We expect new such members to emerge, which were formerly placed close to the Chrysophyceae, but are in fact closely related to Synchromophyceae.

## Supporting Information

S1 Fig Clipping of the 18S rRNA gene alignment. Alignment used for phylogenetic inference (before gblocks treatment) including only the closely related species *S*. *grande*, *C*. *socialis* and *G*. *wildpretii*. Two major insertions are present at the 5’ end (for *C*. *socialis* only; positions 69–106) and the 3’ end (positions 1570–1614 for *C*. *socialis* and *G*. *wildpretii*) compared to *S*. *grande*.
(TIFF)

S2 Fig Secondary structure of 18S rDNA V8 region. Calculated by mfold and visualized by VARNA. Structures inferred for *P*. *flagellatus*, *Leukarachnion* sp. PRA-24, *Ch*. *labyrinthuloides*, *S*. *grande* and *S*. *pusillum* are in concordance with those published for other stramenopiles. *C*. *socialis* and *G*. *wildpretii* possess insertions (grey residues, E45-1) with hypothetical secondary structures which alter the tertiary structure position of helix 46. While structure and sequence of E45-1 do not appear similar, notice that helix 46 (boxed) is identical between *G*. *wildpretii* and *C*. *socialis*, and markedly different from all other analyzed species.
(TIFF)

S3 Fig Distribution of 18S GC-content and length of all eukaryotes and the stramenopiles subgroup. Based on SILVA database entries with sequence quality > 90% and available data in the homologous range of *S*. *grande* CCMP2876 (DQ788730). Medians (red solid lines) and lower and upper quartile (dashed lines) of length and GC-content of the whole dataset are given.
(TIFF)

S4 Fig Phylogenetic trees of stramenopiles using 18S rDNA data. Different models to cope with compositional heterogeneity and rate variation across lineages were used to position *Guanchochroma wildpretii*, based on 78 heterokont taxa with 1506 positions. Formatting identical to Fig 4. (A) 18S Maximum Likelihood subtree (50% consensus) of Synchromophyceae and related organisms under RY-coding with support values for ML and Bayesian Analysis given at nodes. (B) 18S Bayesian Analysis subtree of Synchromophyceae and related organisms under a relaxed clock model. (C) 18S Neighbor Joining subtree using LogDet transformed distances with support values for NJ-LogDet and ML (under a non-homogenous substitution model).
(TIFF)

S5 Fig 18S sequence comparisons between Chryso-, Synuro- and Synchromophyceae. The alignment treated with gblocks (1506 positions) was used. In the lower diagonal uncorrected pairwise distances (p-distance) between sequences A and B are shown. The upper diagonal includes relative rates test of sequence A and B, and outgroup taxon *Nannochloropsis salina* (Eustigmatophyceae) according to Tajima with Holm- Bonferroni correction for multiple comparisons. Significantly different substitution rates given in shades of grey. Sequence numbering according to S1 Table, including *G*. *wildpretii* (77) and *C*. *socialis* (78).
(TIFF)

S6 Fig *Guanchochroma wildpretii*. Reconstruction of cell and lorica development. Model of the formation of multilayered loricae. (A) Migrating amoeba with a nucleus, chloroplasts, mitochondria and vacuolar compartments. A lorica is lacking. Scale bar: 3 μm (B) B1, B2 and Fig 1G present details of the amoeba shown in B3. The amoeba has just settled next to the empty ‘mother’ lorica. The single lorica layer is directly attached to the MCB. The cytoplasm contains single-lobed chloroplasts without protrusions, mitochondria, fragmented vacuoles, multivesicular bodies, and endosymbiontic bacteria in vacuolar compartments. Scale bars B1 and B2: 500 nm; B3: 3 μm. (C) Older sessile amoeba with (bi-)lobed chloroplasts whose thylakoid-free protrusions are grouped in the center of the cell. The MCB is directly attached to the lorica which is composed of an electron-dense outer layer and an inner layer with a loose texture (see insert). Scale bar: 3 μm (D) Detail of Fig 2A. Immediately after binary division, two amoebae with fragmented vacuoles lie within the lorica of the mother cell which is composed of an electron-dense and an electron-lucent layer. The two cells have not yet formed separate new lorica layers of their own. Scale bar: 1 μm (E) Sessile amoeba with a nucleus, large vacuoles, and single-lobed chloroplasts. The MCB is not directly attached to the lorica. Such images might be obtained, if a large portion of the cell´s cytoplasm is used for the formation of reticulopodia joining the meroplasmodial network. Alternatively, this cell represents a sessile amoeba remaining in the mother cell´s lorica just after binary division and migration of the sister cell. The detail E2 shows a mitochondrion and many multivesicular bodies at the cell periphery which are probably involved in the formation of a new lorica layer. Scale bars E1: 3 μm; E2: 500 nm (F) Sessile amoeba with a nucleus, large vacuoles, and single-lobed chloroplasts. The cell has remained in the mother cell´s lorica after binary division and migration of the sister cell. The amoeba does not yet fill the volume of the mother cell´s lorica, but it has already formed a new lorica layer closely adjacent to its MCB. Scale bar: 3 μm (G) Detail of an ostiole. The opening in the outermost lorica layer has been closed by the inner lorica layers which were probably formed after binary cell division by the remaining sessile amoeba. Scale bar: 1 μm (H) Sessile amoeba with a multilayered lorica. Vacuolar or vesicular compartments, filled with an amorphous/fibrillar material lie at the cell periphery and may be involved in the deposition of lorica material. Scale bar: 1 μm (I) Sessile amoeba with a multilayered lorica. The space between the innermost lorica layer and the plasma membrane contains only small amounts of an amorphous/fibrillar material. Note the possibly endosymbiontic bacteria in vacuolar compartments. Scale bar: 1 μm (J) Sessile amoeba with a multilayered lorica. Note the loosely arranged amorphous/fibrillar material between the innermost lorica layer and the plasma membrane, which presumably represents newly deposited lorica material. Scale bar: 1 μm (K) A thick, multilayered lorica encloses a sessile amoeba which possibly functions as a cyst stage. The insert shows an amoeboid stage with two ostioles in the multilayered lorica. The upper ostiole occurs in the outermost lorica layer which does not enclose the entire cell, but is discontinuous on the right side. Here, the second ostiole can be found in the next lorica layer. Most likely, the second ostiole was formed after the deposition of a new lorica layer following binary division and migration of one sister cell. Note that the second ostiole has been closed as well by a new, innermost lorica layer. Scale bar: 3 μm; insert: 3 μm. O—ostiole, L—lorica, mv—multivesicular body, V—vacuole/ vesicle, M—mitochondrium, B—bacterium.
(TIFF)

S7 Fig *Guanchochroma wildpretii*. Chloroplast structure, chloroplast groups and chloroplast membranes I. (A) Details of Fig 1H. A1 and A2 showing bi-lobed chloroplasts in a sessile amoeba besides mitochondria, multivesicular bodies and vacuoles, which are filled with an amorphous/fibrillar material. Serial section analysis of the chloroplasts in A3–A5 confirmed the connectivity of the chloroplast lobes, which are interconnected via plastid sectors lacking thylakoids (asterisk). Since other amoebae (particularly those which are young) possess single-lobed plastids (see panels B2, E1 and F in S6 Fig), the bi-lobed chloroplasts may represent intermediate stages in (synchronized) chloroplast division. Furthermore, the thylakoid-free plastid sectors may become thinner during chloroplast division and may correspond to the thin chloroplast protrusions, which tend to form groups in particular phases of cell development (see Fig 2C; panel 2C in S6 Fig; panel B1 in S7 Fig; panels A1 and B1 in S8 Fig). Scale bars A1–A4: 1 μm; A5: 3 μm. (B) Details of Fig 2C showing the chloroplasts of a sessile amoeba. Their tubular to globular shaped protrusions are grouped in the center of the cell. Each chloroplast is surrounded by four membranes arranged in two membrane pairs in B2 and B3. Scale bars B1: 1 μm; B2 and B3: 100 nm. (C) Although preservation of chloroplast membrane structures is not perfect, there is no indication for the occurrence of true plastid complexes. As expected for single, grouped chloroplasts, the number of membranes between laterally adjacent chloroplast protrusions (bottom) is twice as high as the number of membranes separating a protoplast protrusion from the surrounding cytoplasm (top). Scale bar: 100 nm. Cl—chloroplast lobe, M—mitochondrium, mv—multivesicular body.
(TIFF)

S8 Fig *Guanchochroma wildpretii*. Chloroplast structure, chloroplast groups and chloroplast membranes II. (A) A group of long, narrow chloroplast protrusions is shown in A1. In A2 Regular patterns of small dots often occur in the protrusions. The size of the dots corresponds to 70S ribosoms (20 nm) rather than to ferritin (8–12 nm). The overview A3 shows the grouped chloroplast protrusions in the center of a sessile amoeba. Scale bars: A1, A2: 500nm; A3: 3 μm (B) A group of chloroplast protrusions without dotted patterns. Note the conspicuous constriction in the central protrusion (asterisk) which corresponds to the concept that these structures might represent intermediate stages in chloroplast division. The overview B2 shows the grouped protoplast protrusions in the center of a sessile amoeba. Scale bars: B1: 500nm; B2: 3 μm (C) Details of chloroplast thylakoids which are arranged in stacks of three. Note that the thylakoid stacks are branched and form a continuous network. At the branching points (arrowhead), a thylakoid stack splits up and the single thylakoids aggregate with thylakoids derived from neighboring stacks. Scattered 70S ribosomes and a plastoglobulus can be seen between the thylakoid stacks in C1. Scale bars: 100 nm (D) Detail of chloroplast thylakoids, which are arranged in stacks of three. Two stacks may approach each other closely, so that larger stacks of six thylakoid membranes can also be found. Chloroplasts contain plastoglobuli. Scale bar: 100nm (E) Close detail of chloroplast thylakoids which are arranged in stacks of three. Scale bar: 100nm.
(TIFF)

S9 Fig Subcellular details of *Guanchochroma wildpretii*. (A) Detail of a sessile amoeba whose MCB is not directly attached to the lorica (developmental stage comparable to panel E1 in S6 Fig). The cytoplasm contains the nucleus with a nucleolus, single-lobed chloroplasts, mitochondria, vacuoles with a fibrillar material, and an endosymbiotic bacterium. Scale bar: 500 nm (B) Detail of panel E1 in S6 Fig showing single-lobed chloroplasts, vacuoles, endosymbiotic bacteria, small multivesicular bodies, and a mitochondrium. Scale bar: 500 nm (C) Vacuolar/vesicular compartments in a sessile amoeba. The compartments are filled with a granular/fibrillar material and contain very small vesicles. Scale bar: 500 nm (D) Detail of a large sessile amoeba whose MCB is almost directly attached to the lorica (see insert). Several multivesicular bodies lie at the cell periphery, single-lobed chloroplasts are aligned towards the center of the cell. Mitochondria and endosymbiotic bacteria occur in the cytoplasm besides several vacuoles. Note the conspicuously large electron-dense deposit in one of the vacuoles, which, presumably, is composed of indigestible remnants of food organisms. Scale bar: 1 μm, insert: 3 μm (E) Details of panel F in S6 Fig showing a sessile amoeba after binary division, which has already formed a new lorica layer within the mother cell´s lorica. Part of the nucleus can be seen in the upper left corner of E1. The cytoplasm contains single-lobed chloroplasts, mitochondria, vacuolar and vesicular compartments, and endosymbiotic bacteria. The bacteria undergo cell divisions (arrowhead) within the surrounding vacuoles in E2, which is unlikely for those bacteria that serve as food for the amoeboid cell. Scale bars: 500 nm. B—bacterium, N—nucleus, C—chloroplast, V—vacuole/ vesicle, mv—multivesicular body.
(TIFF)

S10 Fig Subcellular details of *Chrysopodocystis socialis*. (A) Typical sessile amoeba whose thin, single-layered lorica is significantly bigger than the MCB. The detail A2 shows the nucleus with the nucleolus, a single-lobed chloroplast, and two mitochondria. Note that some vacuolar compartments are filled with a homogeneous grey material, while others are electron-lucent. Scale bars A1: 3 μm; A2: 500 nm (B) Sessile amoeba with a thin, single-layered lorica. Multivesicular bodies and single-lobed chloroplast are located at the periphery of the MCB. The nucleus and cisternae of the endoplasmic reticulum are located in the cytoplasm-rich center of the cell. Scale bar: 500 nm (C) A large, multivesicular body is located in the space between the lorica and the plasma membrane of the MCB. Further multivesicular bodies lie at the periphery of the MCB among the single-lobed chloroplasts. The vacuolar compartment is filled with a homogeneous grey material. Scale bar: 500 nm (D) MCB of a sessile amoeba within a bigger, single-layered lorica. Most single-lobed chloroplasts are located laterally at the cell periphery and several roundish vacuoles are arranged around the cytoplasm-rich center of the cell, where the nucleus is situated in another focus plane. The detail C2 shows single-lobed chloroplasts with distinct thylakoid lamellae, vacuoles, a mitochondrion and several multivesicular bodies either located in the cell center or at the cell periphery. Scale bars C1: 3 μm; C2: 500 nm (E) Sessile amoeba with one ostiole in a single-layered lorica and a cross section through the reticulopodium at the mouth of the ostiole (Insert). Sale bar: 5μm (F) Details of the migrating amoeba shown in Fig 3C. The nucleus with the nucleolus can be seen besides single-lobed chloroplasts, mitochondria, small vacuoles and a multivesicular body. Scale bars: 500 nm (G) Detail of a mitochondrium beside a chloroplast of a sessile amoeba. Note the insert showing details of the 4 chloroplast membranes with higher magnification. Scale bar: 200 nm (H) Chloroplasts contain plastoglobuli and thylakoids are arranged in stacks of three or four. The insert shows the four envelope membranes of the chloroplast in higher magnification. Scale bar: 100 nm (I) Overview of sessile amobae. In this section plane, the ostiole is visible in two of the loricae. Scale bar: 3 μm (J) Detail of Fig 3B showing the diaphragm-like structure, which is presumably involved in cytokinesis. Scale bar: 500 nm. L—lorica, V- vacuole/ vesicle, M—mitochondrium, C—chloroplast, N—nucleus, mv—multivesicular body, O—ostiole.
(TIFF)

S11 Fig HPLC analysis of *Guanchochroma wildpretii* pigment extracts. HPLC analysis of *Guanchochroma wildpretii* pigment extracts. Peaks identified as Chlorophylls *c*_1_+*c*_2_ (1), Fucoxanthin (2), Violaxanthin (3), Antheraxanthin (4), Zeaxanthin (5), Chlorophyll *a* (6) and beta-Carotene (7). Representative chromatogram of 3 independent samples.
(TIFF)

S1 Table List of accession numbers of *rbc*L and 18S gene sequences used for phylogenetic inference. (DOCX)

S2 Table Disparity index test and estimated disparity index per site for 18S and *rbc*L gene alignments. (DOCX)

S3 Table Comparison of support values for the *S*. *grande*, *S*. *pusillum* and *Synchroma* clade in phylogenetic analyses under different methods (DOCX)

S1 Text Phylogenetic analysis under compositional heterogeneity and rate variation across lineages. (DOCX)

## References

[1] Rodríguez-Ezpeleta N, Brinkmann H, Burey SC, Roure B, Burger G, Löffelhardt W, et al Monophyly of primary photosynthetic eukaryotes: green plants, red algae, and glaucophytes. Curr Biol. 2005;15: 1325–1330. 10.1016/j.cub.2005.06.040 16051178

[2] Marin B M. Nowack EC, Melkonian M. A plastid in the making: evidence for a second primary endosymbiosis. Protist. 2005;156: 425–432. 10.1016/j.protis.2005.09.001 16310747

[3] Dorrell RG, Howe CJ. What makes a chloroplast? Reconstructing the establishment of photosynthetic symbioses. J Cell Sci. 2012;125: 1865–1875. 10.1242/jcs.102285 22547565

[4] Burki F, Okamoto N, Pombert J-F, Keeling PJ. The evolutionary history of haptophytes and cryptophytes: phylogenomic evidence for separate origins. Proc R Soc B. 2012;279: 2246–2254. 10.1098/rspb.2011.2301 22298847 PMC3321700

[5] Keeling PJ. The number, speed, and impact of plastid endosymbioses in eukaryotic evolution. Annu Rev Plant Biol. 2013;64: 583–607. 10.1146/annurev-arplant-050312-120144 23451781

[6] Parfrey LW, Grant J, Tekle YI, Lasek-Nesselquist E, Morrison HG, Sogin ML, et al Broadly sampled multigene analyses yield a well-resolved eukaryotic tree of life. Syst Biol. 2010;59: 518–533. 10.1093/sysbio/syq037 20656852 PMC2950834

[7] Woehle C, Dagan T, Martin WF, Gould SB. Red and problematic green phylogenetic signals among thousands of nuclear genes from the photosynthetic and Apicomplexa-related *Chromera velia* . Genome Biol Evol. 2011;3: 1220–1230. 10.1093/gbe/evr100 21965651 PMC3205606

[8] Grant J, Tekle YI, Anderson OR, Patterson DJ, Katz LA. Multigene evidence for the placement of a heterotrophic amoeboid lineage *Leukarachnion* sp. among photosynthetic stramenopiles. Protist. 2009;160: 376–385. 10.1016/j.protis.2009.01.001 19282238

[9] Horn S, Ehlers K, Fritzsch G, Gil-Rodríguez MC, Wilhelm C, Schnetter R. *Synchroma grande* spec. nov. (Synchromophyceae class. nov., Heterokontophyta): an amoeboid marine alga with unique plastid complexes. Protist. 2007;158: 277–293. 10.1016/j.protis.2007.02.004 17567535

[10] Přibyl P, Eliáš M, Cepák V, Lukavský J, Kaštánek P. Zoosporogenesis, morphology, ultrastructure, pigment composition, and phylogenetic position of *Trachydiscus minutus* (Eustigmatophyceae, Heterokontophyta). J Phycol. 2012;48: 231–242. 10.1111/j.1529-8817.2011.01109.x 27009667

[11] Schmidt M, Horn S, Flieger K, Ehlers K, Wilhelm C, Schnetter R. *Synchroma pusillum* sp. nov. and other new algal isolates with chloroplast complexes confirm the Synchromophyceae (Ochrophyta) as a widely distributed group of amoeboid algae. Protist. 2012;163: 544–559. 10.1016/j.protis.2011.11.009 22578425

[12] Koch C, Brumme B, Schmidt M, Flieger K, Schnetter R, Wilhelm C. The life cycle of the amoeboid alga *Synchroma grande* (Synchromophyceae, Heterokontophyta)–highly adapted yet equally equipped for rapid diversification in benthic habitats. Plant Biol. 2011;13: 801–808. 10.1111/j.1438-8677.2010.00427.x 21815985

[13] Ettl H. Grundriss der allgemeinen Algologie. Jena: VEB Gustav Fischer Verlag; 1980.

[14] Billard C. *Chrysopodocystis socialis* gen. et sp. nov. (Chrysophyceae) une nouvelle Rhizochrysidale marine loriquée. Bull Soc Bot Fr. 1978;125: 307–312.

[15] Guiry MD, Guiry GM. AlgaeBase. In: World-wide electronic publication, National University of Ireland, Galway [Internet]. 2012. Available: http://www.algaebase.org

[16] Schnetter R, Ruckelshausen U, Seibold G. Mikrospektralphotometrische Untersuchungen über den Entwicklungszyklus von *Ernodesmis verticillata* (Kützing) Børgesen (Siphonocladales, Chlorophyceae). Cryptogamie Algol. 1984;5: 73–78.

[17] Murray MG, Thompson WF. Rapid isolation of high molecular weight plant DNA. Nucl Acids Res. 1980;8: 4321–4325. PMC324241 7433111 10.1093/nar/8.19.4321 PMC324241

[18] Wilhelm C, Volkmar P, Lohmann C, Becker A, Meyer M. The HPLC-aided pigment analysis of phytoplankton cells as a powerful tool in water quality control. Aqua London. 1995;44: 132–141.

[19] Quast C, Pruesse E, Yilmaz P, Gerken J, Schweer T, Yarza P, et al The SILVA ribosomal RNA gene database project: improved data processing and web-based tools. Nucl Acids Res. 2012;41: D590–D596. 10.1093/nar/gks1219 23193283 PMC3531112

[20] R Core Team. R: a language and environment for statistical computing [Internet]. Vienna, Austria: R Foundation for Statistical Computing; 2013 Available: http://www.R-project.org

[21] Charif D, Lobry JR. SeqinR 1.0–2: a contributed package to the R project for statistical computing devoted to biological sequences retrieval and analysis In: Bastolla DU, Porto PDM, Roman DHE, Vendruscolo DM, editors. Structural Approaches to Sequence Evolution. Springer Berlin Heidelberg; 2007 pp. 207–232.

[22] Zuker M. Mfold web server for nucleic acid folding and hybridization prediction. Nucl Acids Res. 2003;31: 3406–3415. 12824337 10.1093/nar/gkg595 PMC169194

[23] Darty K, Denise A, Ponty Y. VARNA: Interactive drawing and editing of the RNA secondary structure. Bioinformatics. 2009;25: 1974–1975. 10.1093/bioinformatics/btp250 19398448 PMC2712331

[24] Van de Peer Y, Caers A, Rijk PD, Wachter RD. Database on the structure of small ribosomal subunit RNA. Nucl Acids Res. 1998;26: 179–182. 10.1093/nar/26.1.179 9399829 PMC147221

[25] De Rijk P, Neefs J-M, Van De Peer Y, De Wachter R. Compilation of small ribosomal subunit RNA sequences. Nucl Acids Res. 1992;20: 2075–2089. 1375995 10.1093/nar/20.suppl.2075 PMC333984

[26] Katoh K, Standley DM. MAFFT multiple sequence alignment software version 7: improvements in performance and usability. Mol Biol Evol. 2013; 10.1093/molbev/mst010 PMC3603318 23329690

[27] Talavera G, Castresana J. Improvement of phylogenies after removing divergent and ambiguously aligned blocks from protein sequence alignments. Syst Biol. 2007;56: 564–577. 10.1080/10635150701472164 17654362

[28] Tamura K, Peterson D, Peterson N, Stecher G, Nei M, Kumar S. MEGA5: molecular evolutionary genetics analysis using Maximum Likelihood, evolutionary distance, and Maximum Parsimony methods. Mol Biol Evol. 2011; 10.1093/molbev/msr121 PMC3203626 21546353

[29] Ronquist F, Teslenko M, van der Mark P, Ayres DL, Darling A, Höhna S, et al MrBayes 3.2: efficient Bayesian Phylogenetic Inference and model choice across a large model space. Syst Biol. 2012;61: 539–542. 10.1093/sysbio/sys029 22357727 PMC3329765

[30] Hall T. BioEdit: a user-friendly biological sequence alignment editor and analysis program for Windows 95/98/NT. Nucl Acid S. 1999;41: 95–98.

[31] Silvestro D, Michalak I. raxmlGUI: a graphical front-end for RAxML. Org Divers Evol. 2012;12: 335–337. 10.1007/s13127-011-0056-0

[32] Stamatakis A. RAxML-VI-HPC: Maximum Likelihood-based phylogenetic analyses with thousands of taxa and mixed models. Bioinformatics. 2006;22: 2688–2690. 10.1093/bioinformatics/btl446 16928733

[33] Tajima F. Simple methods for testing the molecular evolutionary clock hypothesis. Genetics. 1993;135: 599–607. 8244016 10.1093/genetics/135.2.599 PMC1205659

[34] Paradis E. pegas: an R package for population genetics with an integrated–modular approach. Bioinformatics. 2010;26: 419–420. 10.1093/bioinformatics/btp696 20080509

[35] Holm S. A simple sequentially rejective multiple test procedure. Scand J Stat. 1979;6: 65–70.

[36] Knapp S, McNeill J, Turland NJ. Changes to publication requirements made at the XVIII International Botanical Congress in Melbourne—what does e-publication mean for you? BMC Evol Biol. 2011;11: 250 10.1186/1471-2148-11-250 21917189 PMC3173377

[37] Ota S, Ueda K, Ishida K-I. *Lotharella vacuolata* sp. nov., a new species of chlorarachniophyte algae, and time-lapse video observations on its unique post-cell division behavior. Phycological Res. 2005;53: 275–286.

[38] Hirakawa Y, Howe A, James ER, Keeling PJ. Morphological Diversity between Culture Strains of a Chlorarachniophyte, *Lotharella globosa* . PLoS ONE. 2011;6: e23193 10.1371/journal.pone.0023193 21858028 PMC3156133

[39] Ota S, Vaulot D. *Lotharella reticulosa* sp. nov.: a highly reticulated network forming chlorarachniophyte from the Mediterranean Sea. Protist. 2012;163: 91–104. 10.1016/j.protis.2011.02.004 21497132

[40] O’Kelly C, Wujek D. Cell structure and asexual reproduction in *Lagynion delicatulum* (Stylococcaceae, Chrysophyceae). Eur J Phycol. 2001;36: 51–59. 10.1080/09670260110001735198

[41] Brosnan S, Brown PJP, Farmer MA, Triemer RE. Morphological separation of the euglenoid genera *Trachelomonas* and *Strombomonas* (Euglenophyta) based on lorica development and posterior strip reduction. J Phycol. 2005;41: 590–605. 10.1111/j.1529-8817.2005.00068.x

[42] Leadbeater BSC. Choanoflagellate lorica construction and assembly: the nudiform condition. I. *Savillea* species. Protist. 2008;159: 259–268. 10.1016/j.protis.2007.09.005 18162436

[43] Preisfeld A, Berger S, Busse I, Liller S, Ruppel HG. Phylogenetic analyses of various euglenoid taxa (Euglenozoa) based on 18S rDNA sequence data. J Phycol. 2000;36: 220–226. 10.1046/j.1529-8817.2000.99091.x

[44] Von der Heyden S, Chao E, Cavalier-Smith T. Genetic diversity of goniomonads: an ancient divergence between marine and freshwater species. Eur J Phycol. 2004;39: 343–350. 10.1080/09670260400005567

[45] Schweizer M, Pawlowski J, Kouwenhoven TJ, Guiard J, van der Zwaan B. Molecular phylogeny of Rotaliida (Foraminifera) based on complete small subunit rDNA sequences. Mar Micropaleontol. 2008;66: 233–246. 10.1016/j.marmicro.2007.10.003

[46] Galtier N, Piganeau G, Mouchiroud D, Duret L. GC-content evolution in mammalian genomes: the biased gene conversion hypothesis. Genetics. 2001;159: 907–911. 11693127 10.1093/genetics/159.2.907 PMC1461818

[47] Meunier J, Duret L. Recombination drives the evolution of GC-content in the human genome. Mol Biol Evol. 2004;21: 984–990. 10.1093/molbev/msh070 14963104

[48] Eickbush TH, Eickbush DG. Finely orchestrated movements: evolution of the ribosomal RNA genes. Genetics. 2007;175: 477–485. 10.1534/genetics.107.071399 17322354 PMC1800602

[49] Escobar JS, Glémin S, Galtier N. GC-biased gene conversion impacts ribosomal DNA evolution in vertebrates, angiosperms, and other eukaryotes. Mol Biol Evol. 2011;28: 2561–2575. 10.1093/molbev/msr079 21444650

[50] Lopez P, Casane D, Philippe H. Heterotachy, an important process of protein evolution. Mol Biol Evol. 2002;19: 1–7. 11752184 10.1093/oxfordjournals.molbev.a003973

[51] Martin AP, Palumbi SR. Body size, metabolic rate, generation time, and the molecular clock. PNAS. 1993;90: 4087–4091. 10.1073/pnas.90.9.4087 8483925 PMC46451

[52] Davies TJ, Savolainen V, Chase MW, Moat J, Barraclough TG. Environmental energy and evolutionary rates in flowering plants. Proc R Soc Lond B. 2004;271: 2195–2200. 10.1098/rspb.2004.2849 PMC1691837 15475341

[53] Bromham L, Leys R. Sociality and the rate of molecular evolution. Mol Biol Evol. 2005;22: 1393–1402. 10.1093/molbev/msi133 15758201

[54] Lynch M. The origins of genome architecture. Sunderland, Mass: Sinauer Associates; 2007.

[55] McFadden GI, Gilson PR, Waller RF. Molecular phylogeny of chlorarachniophytes based on plastid rRNA and rbcL sequences. Arch Protistenkd. 1995;145: 231–239. 10.1016/S0003-9365(11)80318-0

[56] Škaloud P, Kristiansen J, Škaloudová M. Developments in the taxonomy of silica-scaled chrysophytes—from morphological and ultrastructural to molecular approaches. Nord J Bot. 2013;31: 385–402. 10.1111/j.1756-1051.2013.00119.x

[57] Drouin G, Daoud H, Xia J. Relative rates of synonymous substitutions in the mitochondrial, chloroplast and nuclear genomes of seed plants. Mol Phylogenet Evol. 2008;49: 827–831. 10.1016/j.ympev.2008.09.009 18838124

[58] Hua J, Smith DR, Borza T, Lee RW. Similar relative mutation rates in the three genetic compartments of *Mesostigma* and *Chlamydomonas* . Protist. 2012;163: 105–115. 10.1016/j.protis.2011.04.003 21621456

[59] Smith DR, Hua J, Lee RW, Keeling PJ. Relative rates of evolution among the three genetic compartments of the red alga *Porphyra* differ from those of green plants and do not correlate with genome architecture. Mol Phylogenet Evol. 2012;65: 339–344. 10.1016/j.ympev.2012.06.017 22760027

[60] Smith DR, Keeling PJ. Twenty-fold difference in evolutionary rates between the mitochondrial and plastid genomes of species with secondary red plastids. J Eukaryot Microbiol. 2012;59: 181–184. 10.1111/j.1550-7408.2011.00601.x 22236077

[61] Wei L, Xin Y, Wang D, Jing X, Zhou Q, Su X, et al Nannochloropsis plastid and mitochondrial phylogenomes reveal organelle diversification mechanism and intragenus phylotyping strategy in microalgae. BMC Genomics. 2013;14: 534 10.1186/1471-2164-14-534 23915326 PMC3750441

[62] Grell KG, Heini A, Schuller S. The ultrastructure of *Reticulosphaera socialis* Grell (Heterokontophyta). Eur J Protistol. 1990;26: 37–54. 10.1016/S0932-4739(11)80387-1 23196123

[63] Grell KG. The life-cycle of the marine protist *Reticulosphaera socialis* Grell. Arch Protistenkd. 1989;137: 177–197.

[64] Grell KG. *Reticulosphaera japonensis* n. sp. (Heterokontophyta) from tide pools of the japanese coast. Arch Protistenkd. 1990;138: 257–269.

[65] Murakami R, Hashimoto H. Unusual nuclear division in *Nannochloropsis oculata* (Eustigmatophyceae, Heterokonta) which may ensure faithful transmission of secondary plastids. Protist. 2009;160: 41–49. 10.1016/j.protis.2008.09.002 19013102

[66] Weatherill K, Lambiris I, Pickett-Heaps JD, Deane JA, Beech PL. Plastid division in *Mallomonas* (Synurophyceae, Heterokonta). J Phycol. 2007;43: 535–541. 10.1111/j.1529-8817.2007.00356.x

[67] Miyagishima S. Mechanism of plastid division: from a bacterium to an organelle. Plant Physiol. 2011;155: 1533–1544. 10.1104/pp.110.170688 21311032 PMC3091088

[68] Wenderoth K, Marquardt J, Fraunholz M, Van De Peer Y, Wastl J, Maier U-G. The taxonomic position of *Chlamydomyxa labyrinthuloides* . Eur J Protistol. 1999;34: 97–108. 10.1080/09670269910001736152

