# Supplementary figures and images for: Guanchochroma wildpretii gen. et spec. nov. (Ochrophyta) Provides New Insights into the Diversification and Evolution of the Algal Class Synchromophyceae
Source: PLoS One. 2015 Jul 2;10(7):e0131821. doi: 10.1371/journal.pone.0131821 (PMC4489749; doi:10.1371/journal.pone.0131821)

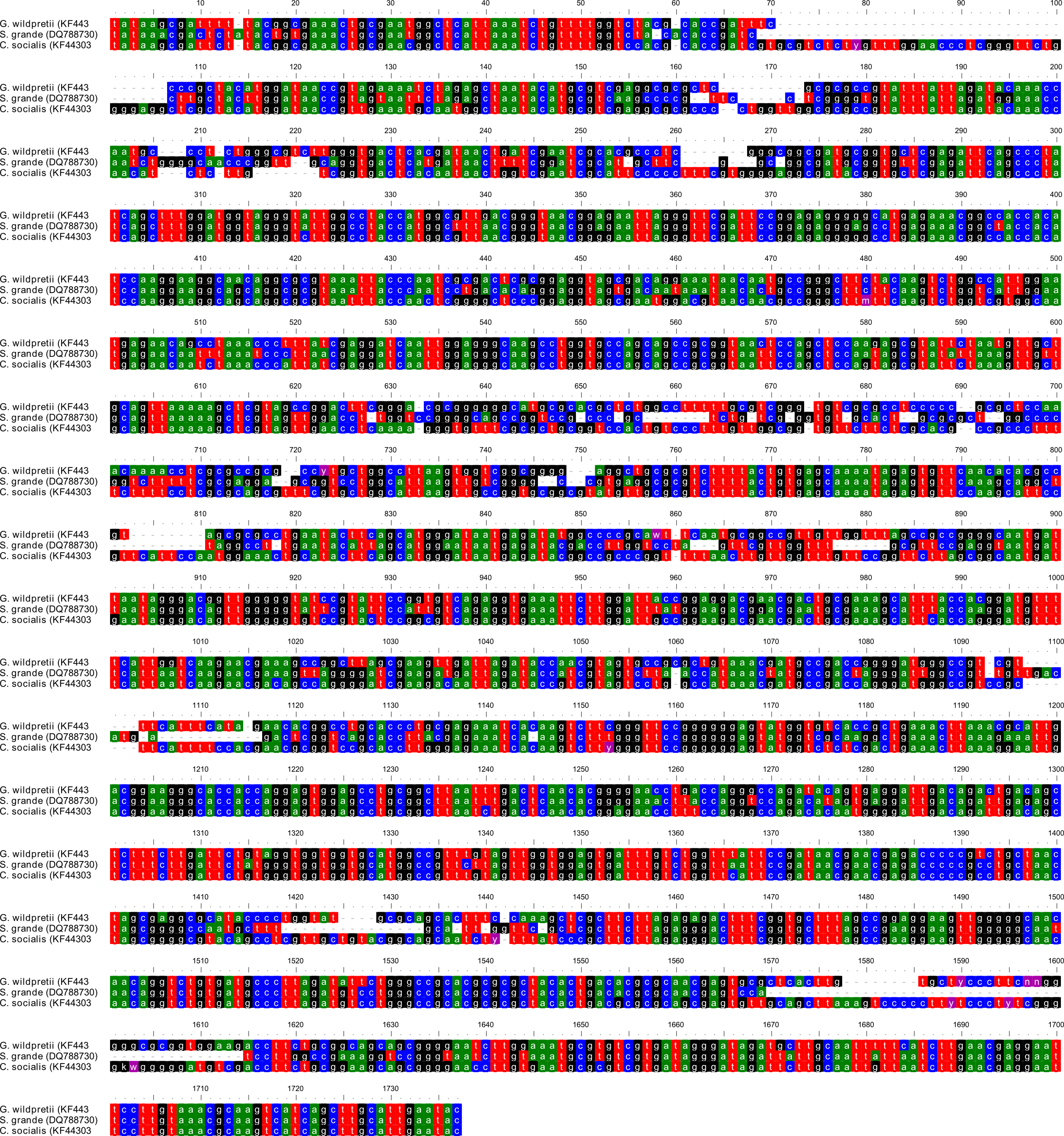

Supplement: S1 Fig — Alignment used for phylogenetic inference (before gblocks treatment) including only the closely related species S. grande, C. socialis and G. wildpretii. Two major insertions are present at the 5’ end (for C. socialis only; positions 69–106) and the 3’ end (positions 1570–1614 for C. socialis and G. wildpretii) compared to S. grande. (TIFF) [file pone.0131821.s001.tiff]

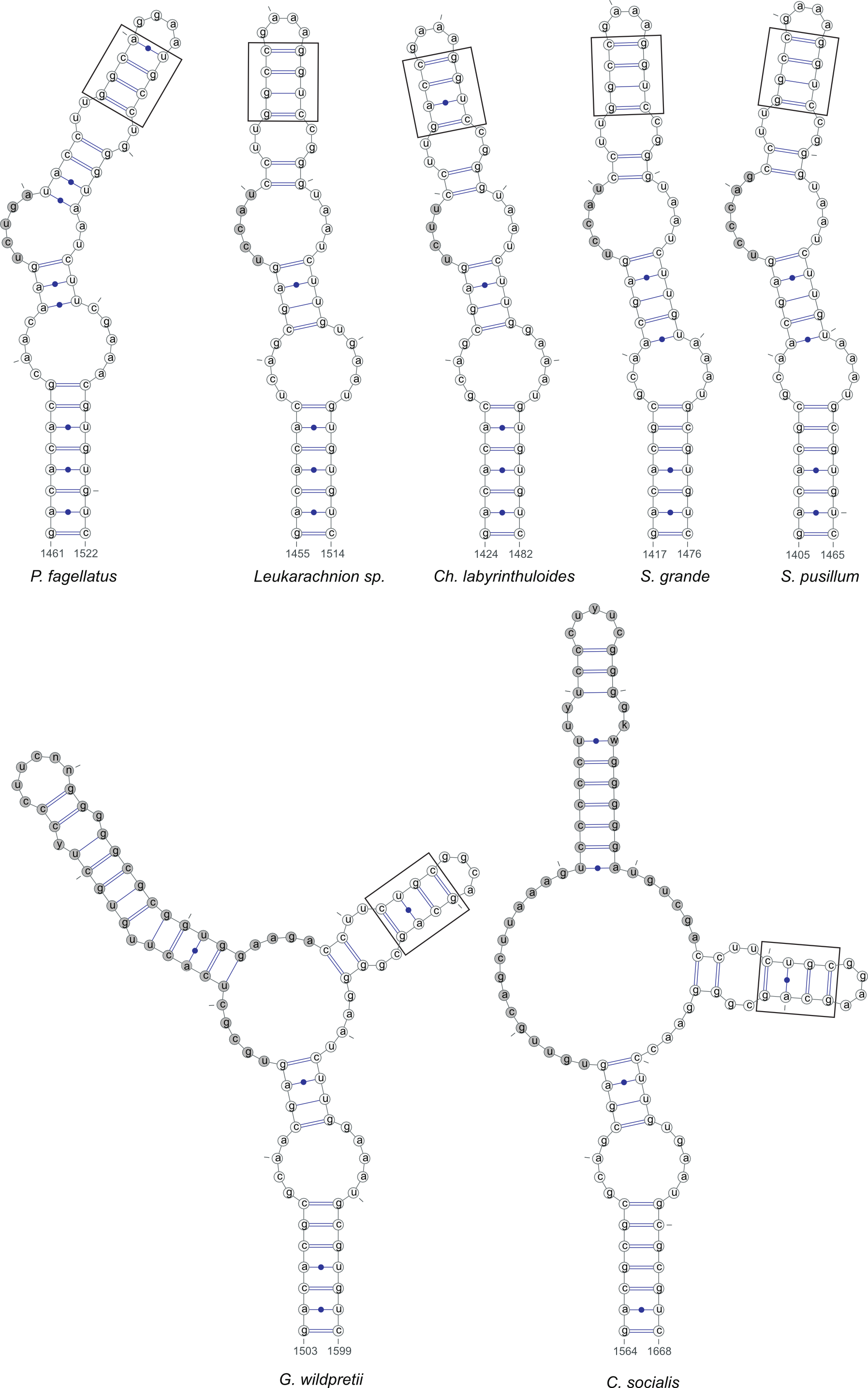

Supplement: S2 Fig — Calculated by mfold and visualized by VARNA. Structures inferred for P. flagellatus, Leukarachnion sp. PRA-24, Ch. labyrinthuloides, S. grande and S. pusillum are in concordance with those published for other stramenopiles. C. socialis and G. wildpretii possess insertions (grey residues, E45-1) with hypothetical secondary structures which alter the tertiary structure position of helix 46. While structure and sequence of E45-1 do not appear similar, notice that helix 46 (boxed) is identical between G. wildpretii and C. socialis, and markedly different from all other analyzed species. (TIFF) [file pone.0131821.s002.tiff]

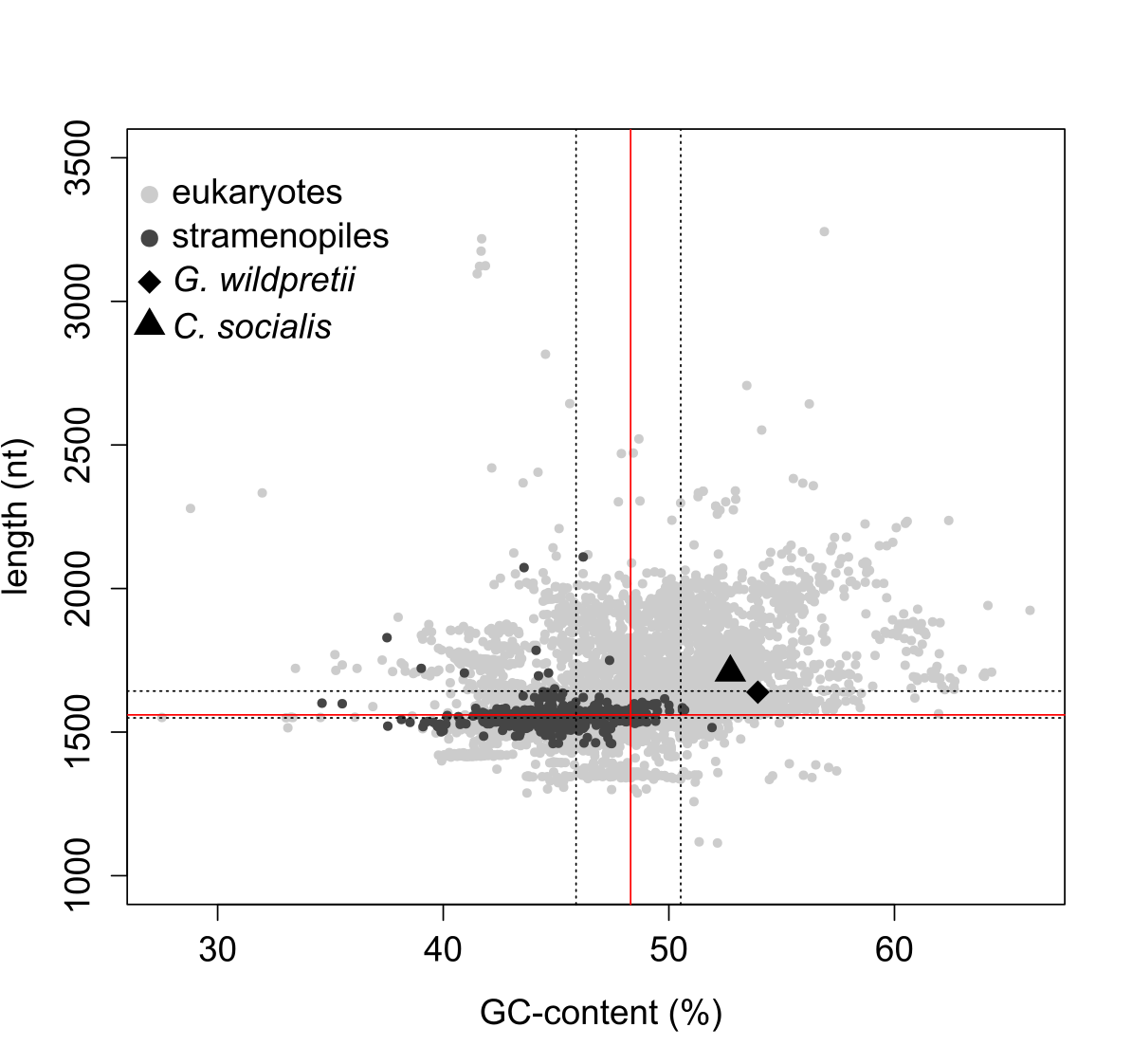

Supplement: S3 Fig — Based on SILVA database entries with sequence quality > 90% and available data in the homologous range of S. grande CCMP2876 (DQ788730). Medians (red solid lines) and lower and upper quartile (dashed lines) of length and GC-content of the whole dataset are given. (TIFF) [file pone.0131821.s003.tiff]

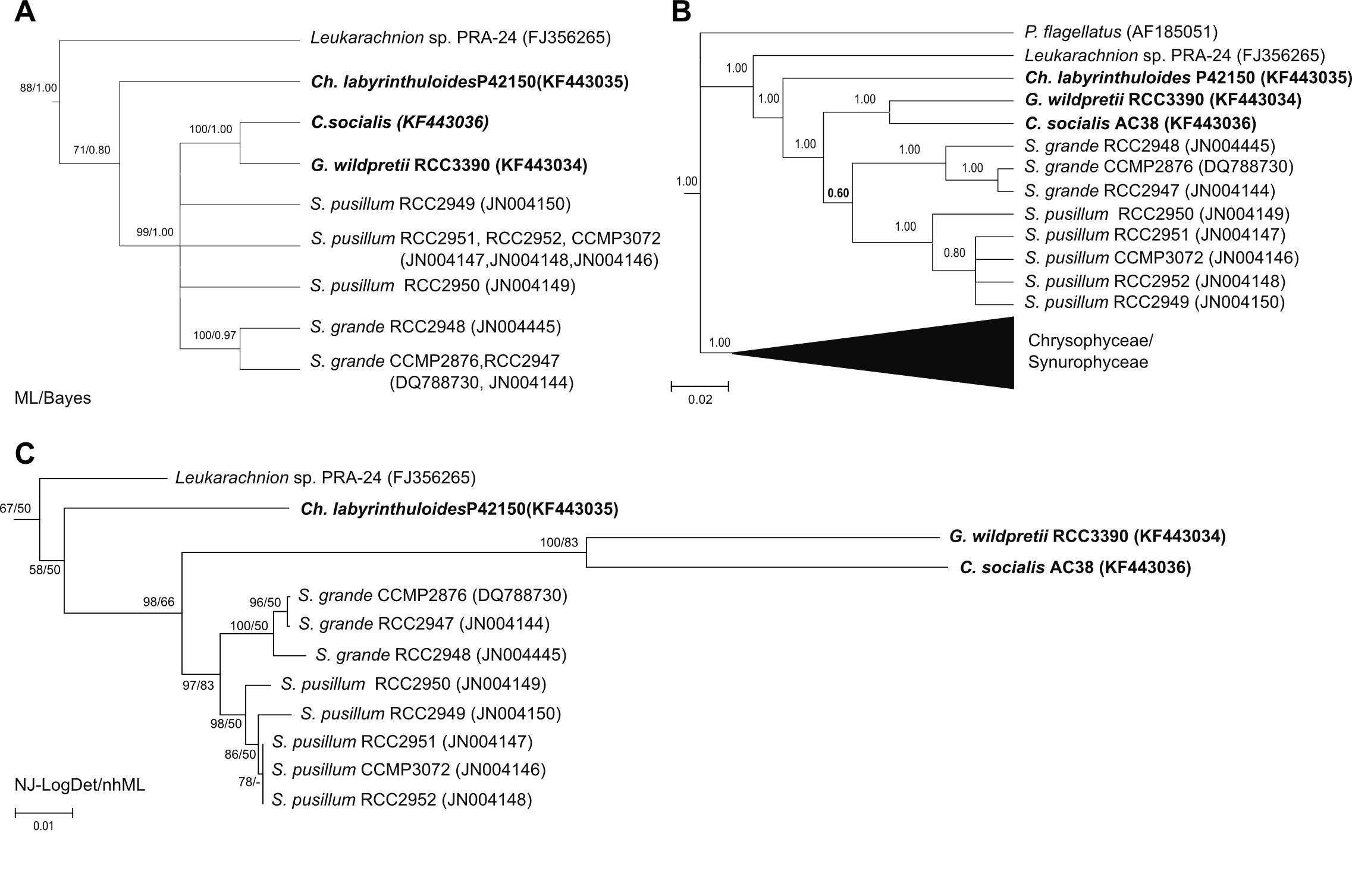

Supplement: S4 Fig — Different models to cope with compositional heterogeneity and rate variation across lineages were used to position Guanchochroma wildpretii, based on 78 heterokont taxa with 1506 positions. Formatting identical to Fig 4. (A) 18S Maximum Likelihood subtree (50% consensus) of Synchromophyceae and related organisms under RY-coding with support values for ML and Bayesian Analysis given at nodes. (B) 18S Bayesian Analysis subtree of Synchromophyceae and related organisms under a relaxed clock model. (C) 18S Neighbor Joining subtree using LogDet transformed distances with support values for NJ-LogDet and ML (under a non-homogenous substitution model). (TIFF) [file pone.0131821.s004.tiff]

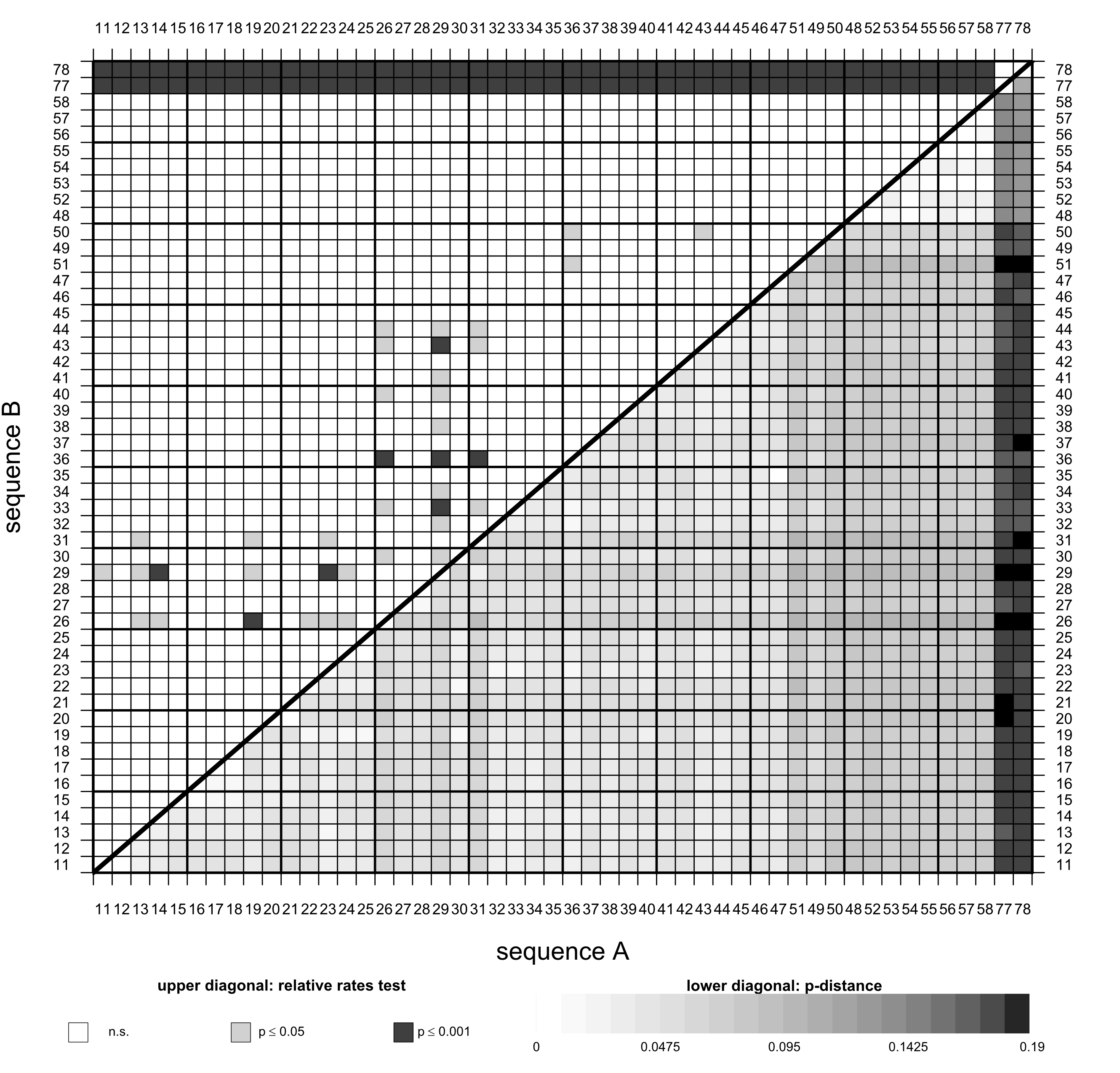

Supplement: S5 Fig — The alignment treated with gblocks (1506 positions) was used. In the lower diagonal uncorrected pairwise distances (p-distance) between sequences A and B are shown. The upper diagonal includes relative rates test of sequence A and B, and outgroup taxon Nannochloropsis salina (Eustigmatophyceae) according to Tajima with Holm- Bonferroni correction for multiple comparisons. Significantly different substitution rates given in shades of grey. Sequence numbering according to S1 Table, including G. wildpretii (77) and C. socialis (78). (TIFF) [file pone.0131821.s005.tiff]

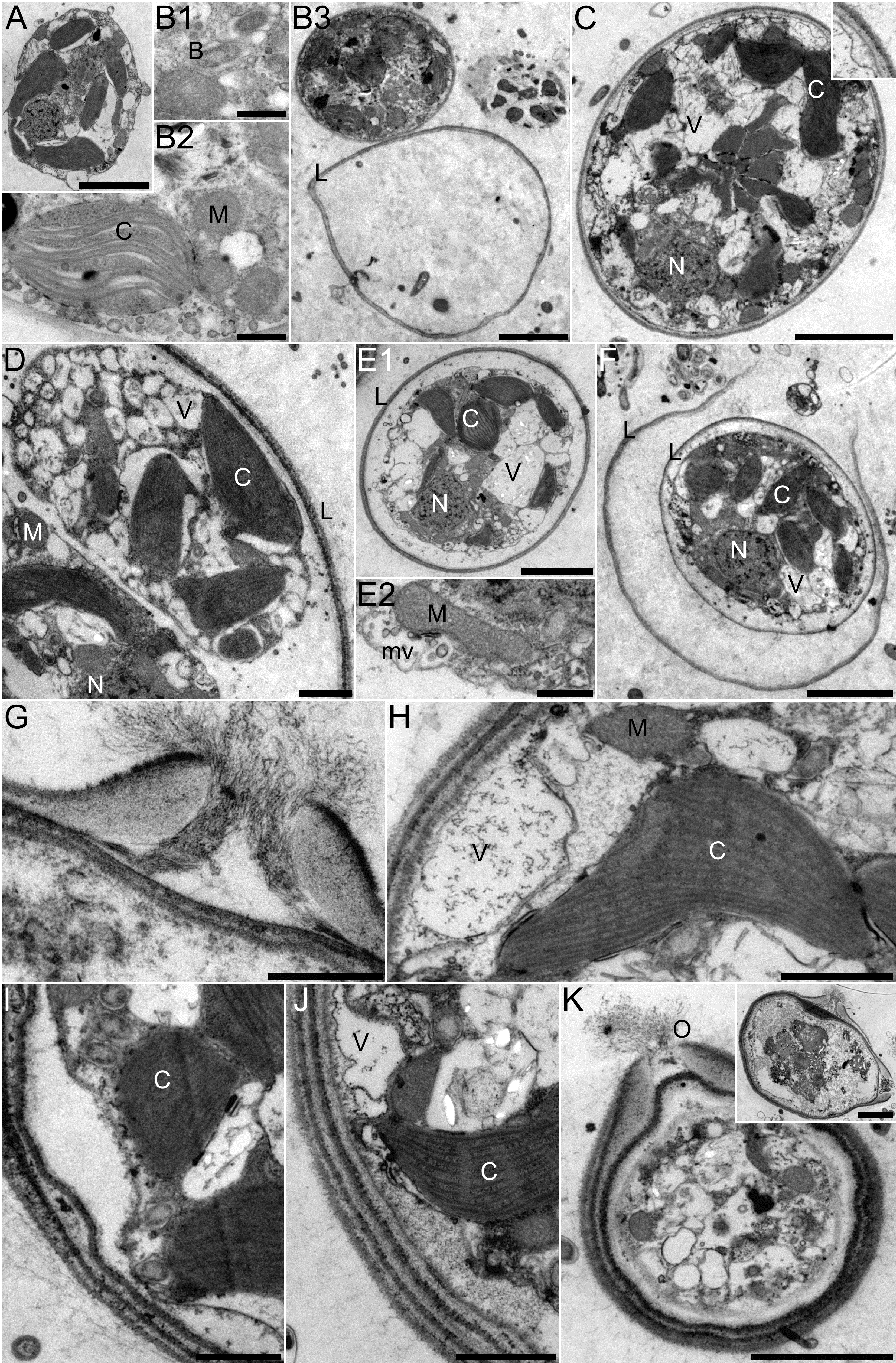

Supplement: S6 Fig — Model of the formation of multilayered loricae. (A) Migrating amoeba with a nucleus, chloroplasts, mitochondria and vacuolar compartments. A lorica is lacking. Scale bar: 3 μm (B) B1, B2 and Fig 1G present details of the amoeba shown in B3. The amoeba has just settled next to the empty ‘mother’ lorica. The single lorica layer is directly attached to the MCB. The cytoplasm contains single-lobed chloroplasts without protrusions, mitochondria, fragmented vacuoles, multivesicular bodies, and endosymbiontic bacteria in vacuolar compartments. Scale bars B1 and B2: 500 nm; B3: 3 μm. (C) Older sessile amoeba with (bi-)lobed chloroplasts whose thylakoid-free protrusions are grouped in the center of the cell. The MCB is directly attached to the lorica which is composed of an electron-dense outer layer and an inner layer with a loose texture (see insert). Scale bar: 3 μm (D) Detail of Fig 2A. Immediately after binary division, two amoebae with fragmented vacuoles lie within the lorica of the mother cell which is composed of an electron-dense and an electron-lucent layer. The two cells have not yet formed separate new lorica layers of their own. Scale bar: 1 μm (E) Sessile amoeba with a nucleus, large vacuoles, and single-lobed chloroplasts. The MCB is not directly attached to the lorica. Such images might be obtained, if a large portion of the cell´s cytoplasm is used for the formation of reticulopodia joining the meroplasmodial network. Alternatively, this cell represents a sessile amoeba remaining in the mother cell´s lorica just after binary division and migration of the sister cell. The detail E2 shows a mitochondrion and many multivesicular bodies at the cell periphery which are probably involved in the formation of a new lorica layer. Scale bars E1: 3 μm; E2: 500 nm (F) Sessile amoeba with a nucleus, large vacuoles, and single-lobed chloroplasts. The cell has remained in the mother cell´s lorica after binary division and migration of the sister cell. The amoeba does not [file pone.0131821.s006.tiff]

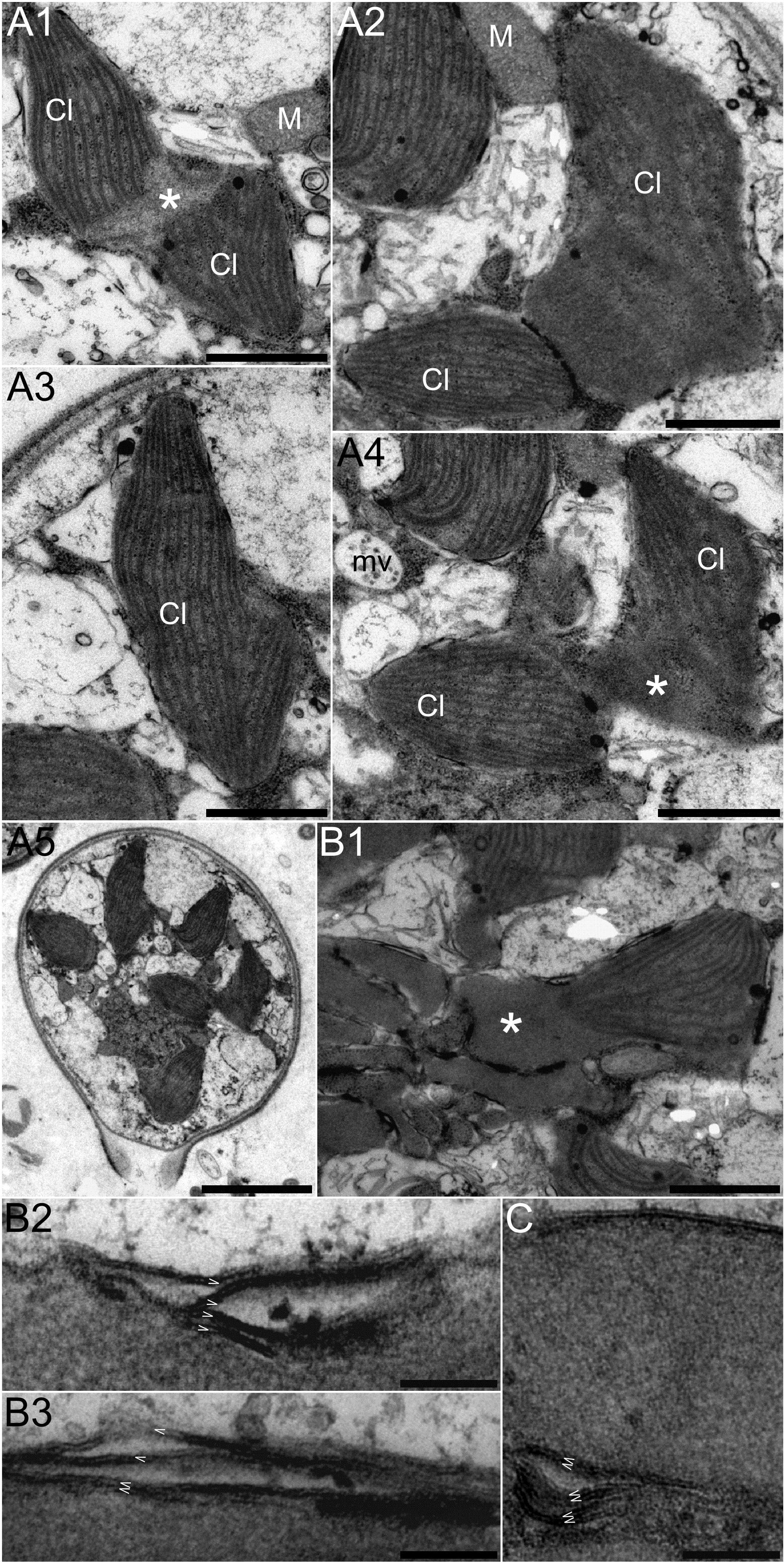

Supplement: S7 Fig — (A) Details of Fig 1H. A1 and A2 showing bi-lobed chloroplasts in a sessile amoeba besides mitochondria, multivesicular bodies and vacuoles, which are filled with an amorphous/fibrillar material. Serial section analysis of the chloroplasts in A3–A5 confirmed the connectivity of the chloroplast lobes, which are interconnected via plastid sectors lacking thylakoids (asterisk). Since other amoebae (particularly those which are young) possess single-lobed plastids (see panels B2, E1 and F in S6 Fig), the bi-lobed chloroplasts may represent intermediate stages in (synchronized) chloroplast division. Furthermore, the thylakoid-free plastid sectors may become thinner during chloroplast division and may correspond to the thin chloroplast protrusions, which tend to form groups in particular phases of cell development (see Fig 2C; panel 2C in S6 Fig; panel B1 in S7 Fig; panels A1 and B1 in S8 Fig). Scale bars A1–A4: 1 μm; A5: 3 μm. (B) Details of Fig 2C showing the chloroplasts of a sessile amoeba. Their tubular to globular shaped protrusions are grouped in the center of the cell. Each chloroplast is surrounded by four membranes arranged in two membrane pairs in B2 and B3. Scale bars B1: 1 μm; B2 and B3: 100 nm. (C) Although preservation of chloroplast membrane structures is not perfect, there is no indication for the occurrence of true plastid complexes. As expected for single, grouped chloroplasts, the number of membranes between laterally adjacent chloroplast protrusions (bottom) is twice as high as the number of membranes separating a protoplast protrusion from the surrounding cytoplasm (top). Scale bar: 100 nm. Cl—chloroplast lobe, M—mitochondrium, mv—multivesicular body. (TIFF) [file pone.0131821.s007.tiff]

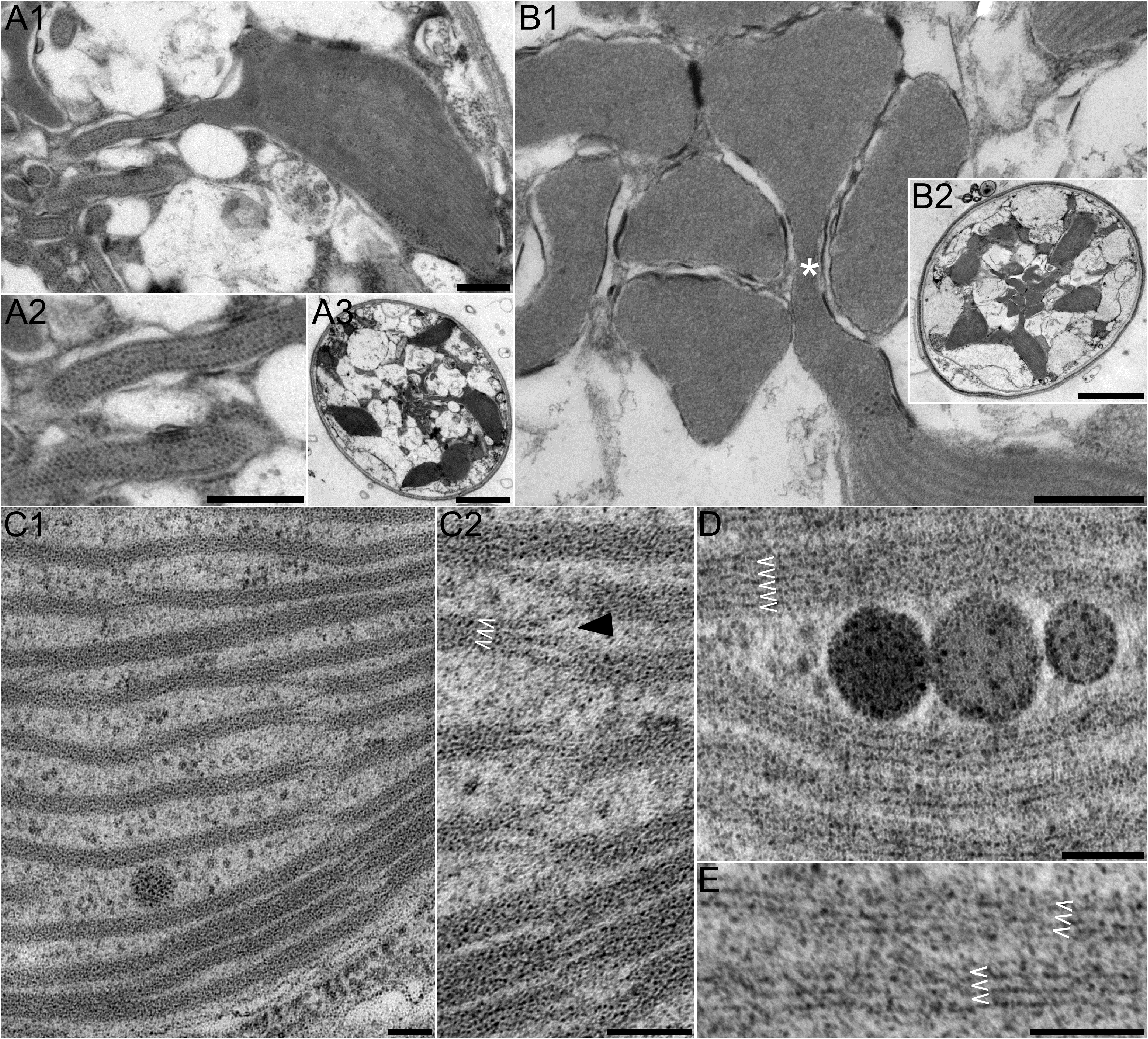

Supplement: S8 Fig — (A) A group of long, narrow chloroplast protrusions is shown in A1. In A2 Regular patterns of small dots often occur in the protrusions. The size of the dots corresponds to 70S ribosoms (20 nm) rather than to ferritin (8–12 nm). The overview A3 shows the grouped chloroplast protrusions in the center of a sessile amoeba. Scale bars: A1, A2: 500nm; A3: 3 μm (B) A group of chloroplast protrusions without dotted patterns. Note the conspicuous constriction in the central protrusion (asterisk) which corresponds to the concept that these structures might represent intermediate stages in chloroplast division. The overview B2 shows the grouped protoplast protrusions in the center of a sessile amoeba. Scale bars: B1: 500nm; B2: 3 μm (C) Details of chloroplast thylakoids which are arranged in stacks of three. Note that the thylakoid stacks are branched and form a continuous network. At the branching points (arrowhead), a thylakoid stack splits up and the single thylakoids aggregate with thylakoids derived from neighboring stacks. Scattered 70S ribosomes and a plastoglobulus can be seen between the thylakoid stacks in C1. Scale bars: 100 nm (D) Detail of chloroplast thylakoids, which are arranged in stacks of three. Two stacks may approach each other closely, so that larger stacks of six thylakoid membranes can also be found. Chloroplasts contain plastoglobuli. Scale bar: 100nm (E) Close detail of chloroplast thylakoids which are arranged in stacks of three. Scale bar: 100nm. (TIFF) [file pone.0131821.s008.tiff]

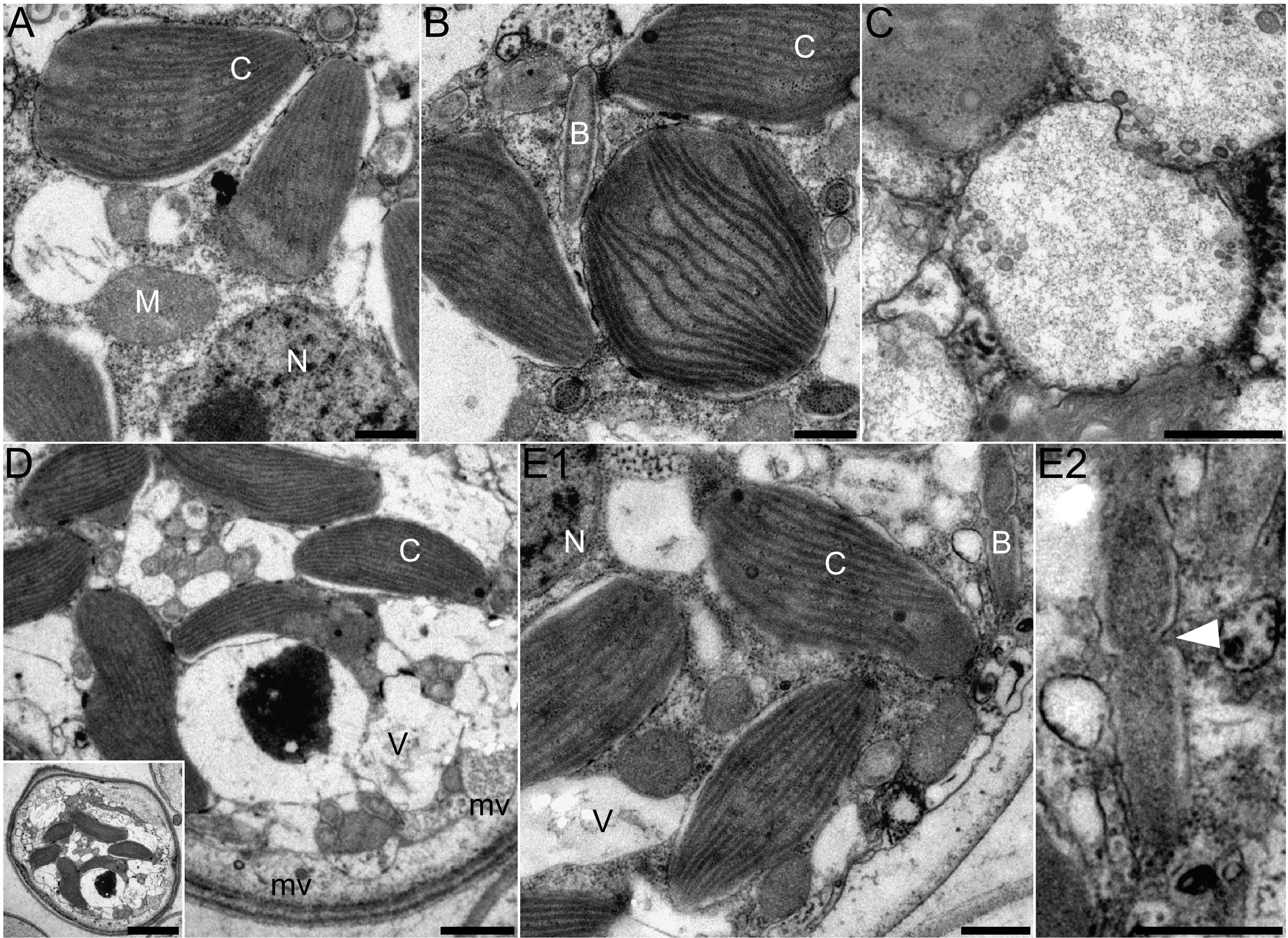

Supplement: S9 Fig — (A) Detail of a sessile amoeba whose MCB is not directly attached to the lorica (developmental stage comparable to panel E1 in S6 Fig). The cytoplasm contains the nucleus with a nucleolus, single-lobed chloroplasts, mitochondria, vacuoles with a fibrillar material, and an endosymbiotic bacterium. Scale bar: 500 nm (B) Detail of panel E1 in S6 Fig showing single-lobed chloroplasts, vacuoles, endosymbiotic bacteria, small multivesicular bodies, and a mitochondrium. Scale bar: 500 nm (C) Vacuolar/vesicular compartments in a sessile amoeba. The compartments are filled with a granular/fibrillar material and contain very small vesicles. Scale bar: 500 nm (D) Detail of a large sessile amoeba whose MCB is almost directly attached to the lorica (see insert). Several multivesicular bodies lie at the cell periphery, single-lobed chloroplasts are aligned towards the center of the cell. Mitochondria and endosymbiotic bacteria occur in the cytoplasm besides several vacuoles. Note the conspicuously large electron-dense deposit in one of the vacuoles, which, presumably, is composed of indigestible remnants of food organisms. Scale bar: 1 μm, insert: 3 μm (E) Details of panel F in S6 Fig showing a sessile amoeba after binary division, which has already formed a new lorica layer within the mother cell´s lorica. Part of the nucleus can be seen in the upper left corner of E1. The cytoplasm contains single-lobed chloroplasts, mitochondria, vacuolar and vesicular compartments, and endosymbiotic bacteria. The bacteria undergo cell divisions (arrowhead) within the surrounding vacuoles in E2, which is unlikely for those bacteria that serve as food for the amoeboid cell. Scale bars: 500 nm. B—bacterium, N—nucleus, C—chloroplast, V—vacuole/ vesicle, mv—multivesicular body. (TIFF) [file pone.0131821.s009.tiff]

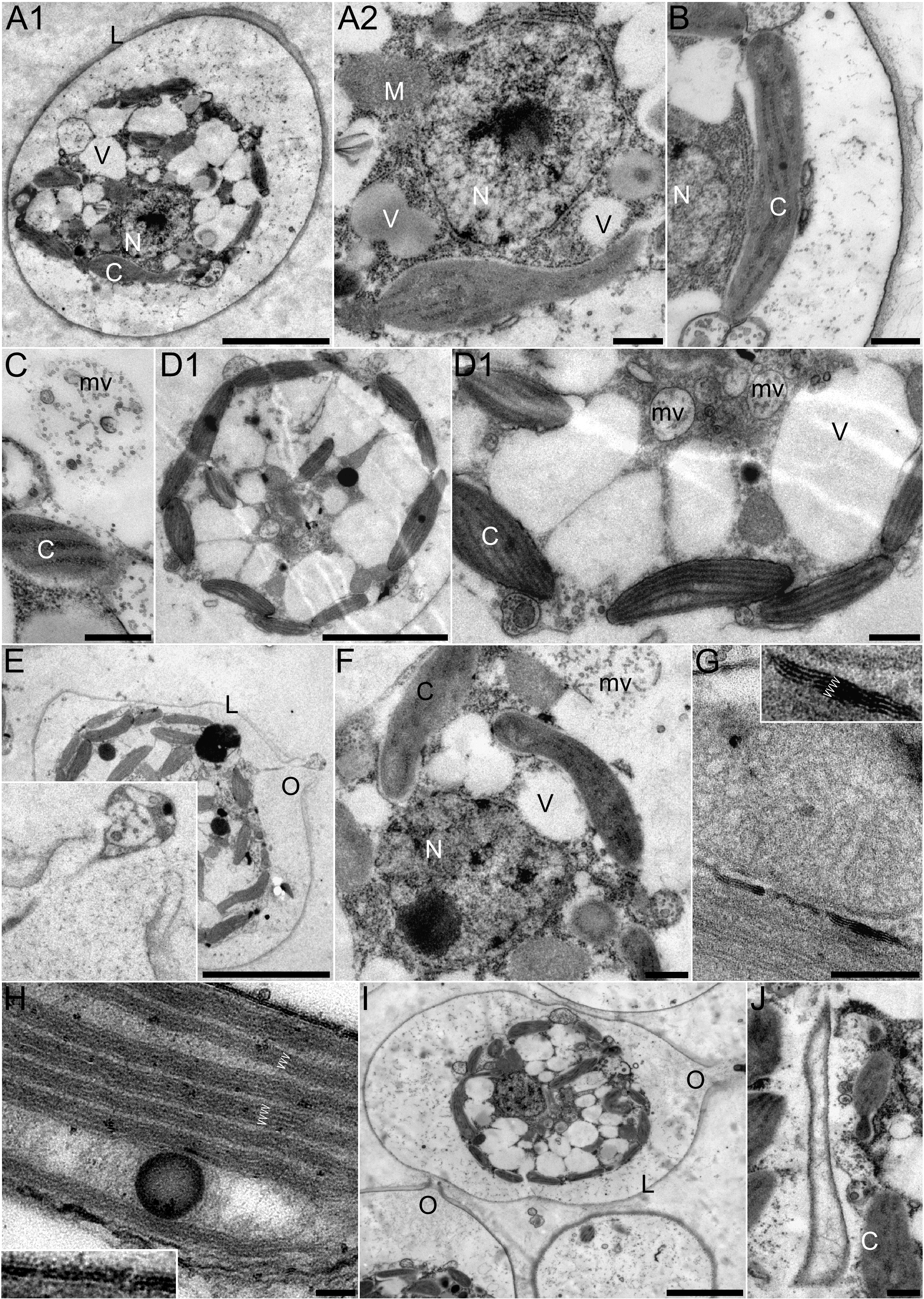

Supplement: S10 Fig — (A) Typical sessile amoeba whose thin, single-layered lorica is significantly bigger than the MCB. The detail A2 shows the nucleus with the nucleolus, a single-lobed chloroplast, and two mitochondria. Note that some vacuolar compartments are filled with a homogeneous grey material, while others are electron-lucent. Scale bars A1: 3 μm; A2: 500 nm (B) Sessile amoeba with a thin, single-layered lorica. Multivesicular bodies and single-lobed chloroplast are located at the periphery of the MCB. The nucleus and cisternae of the endoplasmic reticulum are located in the cytoplasm-rich center of the cell. Scale bar: 500 nm (C) A large, multivesicular body is located in the space between the lorica and the plasma membrane of the MCB. Further multivesicular bodies lie at the periphery of the MCB among the single-lobed chloroplasts. The vacuolar compartment is filled with a homogeneous grey material. Scale bar: 500 nm (D) MCB of a sessile amoeba within a bigger, single-layered lorica. Most single-lobed chloroplasts are located laterally at the cell periphery and several roundish vacuoles are arranged around the cytoplasm-rich center of the cell, where the nucleus is situated in another focus plane. The detail C2 shows single-lobed chloroplasts with distinct thylakoid lamellae, vacuoles, a mitochondrion and several multivesicular bodies either located in the cell center or at the cell periphery. Scale bars C1: 3 μm; C2: 500 nm (E) Sessile amoeba with one ostiole in a single-layered lorica and a cross section through the reticulopodium at the mouth of the ostiole (Insert). Sale bar: 5μm (F) Details of the migrating amoeba shown in Fig 3C. The nucleus with the nucleolus can be seen besides single-lobed chloroplasts, mitochondria, small vacuoles and a multivesicular body. Scale bars: 500 nm (G) Detail of a mitochondrium beside a chloroplast of a sessile amoeba. Note the insert showing details of the 4 chloroplast membranes with higher magnification. Scale bar: 200 nm (H) Chloropla [file pone.0131821.s010.tiff]

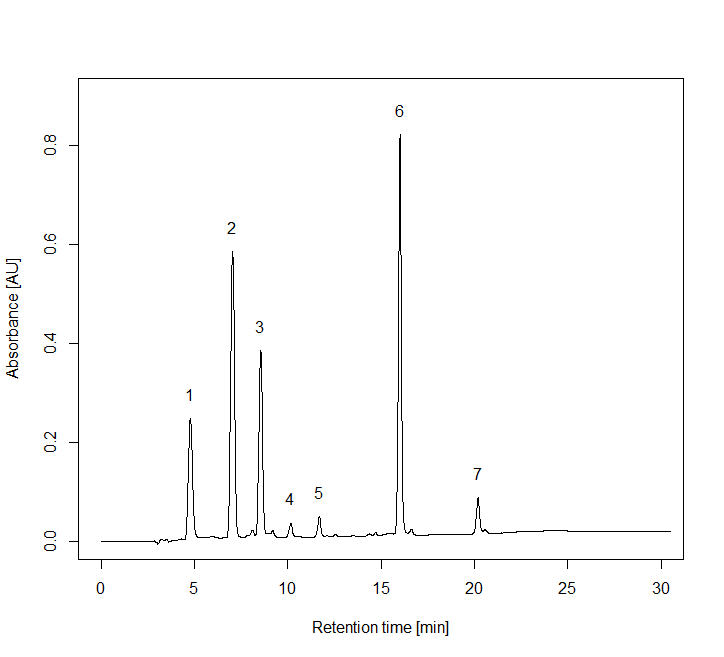

Supplement: S11 Fig — HPLC analysis of Guanchochroma wildpretii pigment extracts. Peaks identified as Chlorophylls c 1+c 2 (1), Fucoxanthin (2), Violaxanthin (3), Antheraxanthin (4), Zeaxanthin (5), Chlorophyll a (6) and beta-Carotene (7). Representative chromatogram of 3 independent samples. (TIFF) [file pone.0131821.s011.tiff]
