# Supplementary material for: Guanchochroma wildpretii gen. et spec. nov. (Ochrophyta) Provides New Insights into the Diversification and Evolution of the Algal Class Synchromophyceae
Source: PLoS One. 2015 Jul 2;10(7):e0131821. doi: 10.1371/journal.pone.0131821 (PMC4489749; doi:10.1371/journal.pone.0131821)
Supplement: S1 Table — (DOCX) [file pone.0131821.s012.docx]

**S1 Table** List of accession numbers of *rbc*L and 18S genes used for phylogenetic inference.

|  | **class** | **rbcL** | **18S** | **species** | **strain** |
| --- | --- | --- | --- | --- | --- |
| 1 | Pinguiophyceae | AF438320 | AB058925 | *Polypodochrysis teissieri* | MBIC10541 |
| 2 | Pinguiophyceae | AF438317 | AB058926 | *Pinguiochrysis pyriformis* | MBIC10872 |
| 3 | Pinguiophyceae | AF438318 | AF438325 | *Glossomastix chrysoplasta* | CCMP1537 |
| 4 | Pinguiophyceae | AF438319 | AF438324 | *Pinguiococcus pyrenoidosus* | CCMP1144 |
| 5 | Eustigmatophyceae | AB280615 | U41051 | *Eustigmatos magnus* | Iida et al. 2007 |
| 6 | Eustigmatophyceae | AM421007 | AF045051 | *Vischeria helvetica* | SAG 876-1 |
| 7 | Eustigmatophyceae | AB052281 | AB052273 | *Nannochloropsis oceanica* | MBIC10090 |
| 8 | Eustigmatophyceae | AB052287 | AB052278 | *Nannochloropsis salina* | MBIC10063 |
| 9 | Chrysophyceae | EF165160 | AF123296 | *Phaeoplaca thallosa* | CCMP634 |
| 10 | Chrysophyceae | EF165158 | EF165141 | *Dinobryon sociale* | UTCC392 |
| 11 | Chrysophyceae | EF165157 | EF165140 | *Dinobryon cylindricum* | CCMP2766 |
| 12 | Chrysophyceae | AF015571 | AF123298 | *Epipyxis pulchra* | CCMP382 |
| 13 | Chrysophyceae | EF165155 | AF123301 | *Epipyxis aureus* | CCMP385 |
| 14 | Chrysophyceae | EF165167 | EF165120 | *Chrysosaccus* sp. | CCMP1156 |
| 15 | Chrysophyceae | EF165166 | EF165121 | *Chrysosaccus* sp. | CCMP368 |
| 16 | Chrysophyceae | EF165164 | EF165106 | *Chromophyton* cf. *rosanoffii* | CCMP2751 |
| 17 | Chrysophyceae | EF165165 | EF165107 | *Chromophyton* cf. *rosanoffii* | CCMP2753 |
| 18 | Chrysophyceae | AF015572 | M87331 | *Hibberdia magna* | CCMP453 |
| 19 | Chrysophyceae | EF165162 | AF123288 | *Lagynion scherffelii* | CCMP465 |
| 20 | Chrysophyceae | EF165161 | EF165146 | *Lagynion* cf. *ampullaceum* | CCMP2727 |
| 21 | Chrysophyceae | EF165150 | EF165104 | *Chrysonebula flava* | CCMP2765 |
| 22 | Chrysophyceae | AF015570 | AF123297 | *Chrysolepidomonas dendrolepidota* | CCMP293 |
| 23 | Chrysophyceae | EF165149 | EF165145 | *Chrysocapsa paludosa* | CCMP380 |
| 24 | Chrysophyceae | EF165148 | EF165105 | *Chrysocapsa vernalis* | CCMP278 |
| 25 | Chrysophyceae | EF165154 | AF123284 | *Naegeliella flagellifera* | CCMP280 |
| 26 | Chrysophyceae | EF165182 | AF123287 | *Chrysamoeba mikrokonta* | CCMP1857 |
| 27 | Chrysophyceae | EF165181 | EF165102 | *Chrysamoeba tenera* | UTCC273 |
| 28 | Chrysophyceae | AF155876 | AF123285 | *Chromulina nebulosa* | CCMP263 |
| 29 | Chrysophyceae | EF165151 | EF165103 | *Chromulina* sp. | SAG17.97 |
| 30 | Chrysophyceae | EF165180 | EF165101 | *Chromulina* cf. *nebulosa* | CCMP2719 |
| 31 | Chrysophyceae | EF165176 | AF123302 | *Chrysoxys* sp. | CCMP591 |
| 32 | Chrysophyceae | EF185314 | EF165142 | *Ochromonas* sp. | CCMP1393 |
| 33 | Chrysophyceae | EF185315 | AF123293 | *Ochromonas tuberculata* | CCMP1861 |
| 34 | Chrysophyceae | EF165171 | EF165113 | *Ochromonas cf. gloeopara* | CCMP2060 |
| 35 | Chrysophyceae | EF165203 | EF165138 | *Ochromonas marina* | AC22 |
| 36 | Chrysophyceae | EF165187 | EF165143 | *Ochromonas perlata* | CCMP2732 |
| 37 | Chrysophyceae | EF165179 | AF123290 | *Uroglena americana* | CCMP1863 |
| 38 | Synurophyceae | EF165198 | EF165118 | *Mallomonas insignis* | CCMP2549 |
| 39 | Synurophyceae | EF165193 | EF165127 | *Mallomonas annulata* | CCMP474 |
| 40 | Synurophyceae | GU325412 | EF469638 | *Mallomonas caudata* | AKC |
| 41 | Synurophyceae | EF165197 | U73221 | *Synura sphagnicola* | CCMP1705 |
| 42 | Synurophyceae | EF165196 | EF165128 | *Synura curtispina* | CCMP847 |
| 43 | Synurophyceae | EF165192 | U73222 | *Synura uvella* | CCMP871 |
| 44 | Synurophyceae | EF165189 | EF165116 | *Synura petersenii* | CCMP854 |
| 45 | Synurophyceae | EF165199 | EF165119 | *Tessellaria volvocina* | CCMP1781 |
| 46 | Synurophyceae | EF165169 | EF165114 | *Poterioochromonas malhamensis* | SAG933.1c |
| 47 | Synchromophyceae | DQ788731 | DQ788730 | *Synchroma grande* | CCMP2876, RCC2946 |
| 48 |  | --- | FJ356265 | *Leukarachnion* sp. | ATCC PRA-24 |
| 49 |  | KF443038 | KF443035 | *Chlamydomyxa labyrinthuloides* | P42150 (CCAM) |
| 50 |  | --- | AF185051 | *Picophagus flagellatus* | RCC 22 |
| 51 | Synchromophyceae | JN004152 | JN004145 | *Synchroma grande* | RCC2948 |
| 52 | Synchromophyceae | JN004154 | JN004147 | *Synchroma pusillum* | RCC2951 |
| 53 | Synchromophyceae | JN004151 | JN004144 | *Synchroma grande* | RCC2947 |
| 54 | Synchromophyceae | JN004153 | JN004146 | *Synchroma pusillum* | CCMP3072,  RCC 2953 |
| 55 | Synchromophyceae | JN004156 | JN004149 | *Synchroma pusillum* | RCC2950 |
| 56 | Synchromophyceae | JN004155 | JN004148 | *Synchroma pusillum* | RCC2952 |
| 57 | Synchromophyceae | JN004157 | JN004150 | *Synchroma pusillum* | RCC2949 |
| 58 | Oomycota | --- | AJ238663 | *Apodachlya brachynema* | 5001a |
| 59 | Bolidophyceae | AF333977 | AF123596 | *Bolidomonas mediterranea* | MINB11E5 |
| 60 | Bacillariophyceae | HQ912419 | AAFD02000029 | *Thalassiosira pseudonana* | CCMP1335 |
| 61 | Xanthophyceae | AJ874340 | AM490824 | *Tribonema minus* | SAG 880-3 |
| 62 | Xanthophyceae | AF064744 | AF083400 | *Mischococcus sphaerocephalus* | UTEX 150 |
| 63 | Haptophyta | AY741371 | L04957 | *Emiliania huxleyi* | CCMP 373 |
| 64 | Oomycota | --- | AJ238656 | *Achlya apiculata* | Dick et al. 1999 |
| 65 | Bolidophyceae | HQ912421 | AF123595 | *Bolidomonas pacifica* | CCMP1866 |
| 66 | Bacillariophyceae | HQ912428 | HQ912564 | *Odontella sinensis* | CCMP1815 |
| 67 | Raphidophyceae | EU168190 | DQ470658 | *Heterosigma akashiwo* | NIES 293 |
| 68 | Raphidophyceae | AF015581 | U41649 | *Chattonella subsalsa* | CCMP217 |
| 69 | Pelagophyceae | AF117906 | AF118443 | *Aureococcus anophagefferens* | CCMP1784 |
| 70 | Dictyochophyceae | AB280611 | U14388 | *Rhizochromulina sp.* | CCMP237 |
| 71 | Dictyochophyceae | U89899 | U14387 | *Pseudopedinella elastica* | CCMP716 |
| 72 | Schizocladiophyceae | AB085615 | AB085614 | *Schizocladia ischiensis* | Kawai et al. 2003 |
| 73 | Phaeophyceae | AY307407 | AY307395 | *Bodanella lauterborni* | UTEX LB 2190 |
| 74 | Phaeophyceae | AY307410 | AY307398 | *Ectocarpus siliculosus* | 3477 |
| 75 | Chrysomerophyceae | AB365205 | AB365204 | *Giraudyopsis* sp. | NIES-1862 |
| 76 | Aurearenophyceae | AB365193 | AB365192 | *Aurearena cruciata* | NIES-1863 |
| 77 |  | KF443039 | KF443036 | *Chrysopodocystis socialis* | AC38 |
| 78 |  | KF443037 | KF443034 | *Guanchochroma wildpretii* | RCC3390 |
