# Supplementary material for: Guanchochroma wildpretii gen. et spec. nov. (Ochrophyta) Provides New Insights into the Diversification and Evolution of the Algal Class Synchromophyceae
Source: PLoS One. 2015 Jul 2;10(7):e0131821. doi: 10.1371/journal.pone.0131821 (PMC4489749; doi:10.1371/journal.pone.0131821)
Supplement: S2 Table — (DOCX) [file pone.0131821.s013.docx]

**S2 Table.** Disparity index test and estimated disparity index per site for 18S and *rbc*L gene alignments, comparing *G. wildpretii* and *C. socialis* to other heterokont higher order taxa or organisms with ambiguous classification. P-values ≤ 0.05 for the disparity index test indicate significant differences in substitution pattern and are marked by * and the percentage of isolates of that group for which this holds true is given.

| Taxon/ organism (no. of isolates) | **18S**  Mean I_d_ per site (I_d_ test) | | **rbcL**  Mean I_d_ per site (I_d_ test) | |
| --- | --- | --- | --- | --- |
|  | *C. socialis* | *G. wildpretii* | *C. socialis* | *G. wildpretii* |
| Aurearenophyceae (1) | 0.346 (* 100%) | 0.537 (* 100%) | 0.145 | 0.560 (* 100%) |
| Bacillariophyceae (2) | 3.120 (* 100%) | 3.651 (* 100%) | 0.101 | 0.073 |
| Bolidophyceae (2) | 4.310 (* 100%) | 4.440 (* 100%) | 0.272 (* 50%) | 0.129 |
| Chrysomerophyceae (1) | 3.200 (* 100%) | 3.677 (* 100%) | 0.079 | 0.000 |
| Chryso-/ Synurophyceae (38) | 4.340 (* 100%) | 4.497 (* 100%) | 0.889 (* 89%) | 0.347 (* 47%) |
| Dictyochophyceae (2) | 2.510 (* 100%) | 2.820 (* 100%) | 0.224 | 0.856 (* 100%) |
| Eustigmatophyceae (4) | 2.550 (* 100%) | 3.040 (* 100%) | 0.212(* 50%) | 0.344 (* 50%) |
| Oomycetes (2) | 5.000 (* 100%) | 4.980 (* 100%) | --- | --- |
| Pelagophyceae (1) | 1.433 (* 100%) | 1.845 (* 100%) | 0.216 | 1.025 (* 100%) |
| Phaeophyceae (2) | 1.830 (* 100%) | 2.190 (* 100%) | 0.109 | 0.000 |
| Pinguiophyceae (4) | 2.380 (* 100%) | 2.820 (* 100%) | 0.073 | 0.370 (* 50%) |
| Raphidophyceae (2) | 3.230 (* 100%) | 3.510 (* 100%) | 0.054 | 0.425 (* 50%) |
| Schizocladiophyceae (1) | 1.196 (* 100%) | 1.430 (* 100%) | 0.498 (* 100%) | 0.000 |
| Synchromophyceea (8) | 1.290 (* 100%) | 1.730 (* 100%) | 0.135 (* 12%) | 0.000 |
| Xanthophyceae (2) | 1.450 (* 100%) | 2.060 (* 100%) | 0.269 | 0.372 (* 50%) |
| *C. socialis* |  | **0.000** |  | **0.043** |
| *Ch. labyrinthuloides* | 2.439 (* 100%) | 2.781 (* 100%) | 1.138 (* 100%) | 0.355 (* 100% |
| *Emiliania huxleyi* | 0.625 (* 100%) | 1.232 (* 100%) | 0.743 (* 100% ) | 1.725 (* 100%) |
| *Leukarachnion* sp. PRA-24 | 2.523 (* 100%) | 3.038 (* 100%) | --- | --- |
| *P. flagellatus* | 2.374 (* 100%) | 2.684 (* 100%) | --- | --- |
