# Supplementary material for: Guanchochroma wildpretii gen. et spec. nov. (Ochrophyta) Provides New Insights into the Diversification and Evolution of the Algal Class Synchromophyceae
Source: PLoS One. 2015 Jul 2;10(7):e0131821. doi: 10.1371/journal.pone.0131821 (PMC4489749; doi:10.1371/journal.pone.0131821)
Supplement: S3 Table — (DOCX) [file pone.0131821.s014.docx]

**S3 Table.** Comparison of support values for the *S. grande*, *S. pusillum* and *Synchroma* clade in phylogenetic analyses under different methods (ML, NJ, MP and Bayesian analysis) with complete (all) or reduced (no *G.w.*, no *C.s.*) *rbc*L and 18S dataset, leaving all other parameters static. Bayesian analysis results under a relaxed clock assumption are given in parentheses. Additionally, results are given for the datasets excluding both *G. wildpretii* and *C. socialis* either from this study (Bayesian) or Schmidt et al. (2012) (ML, NJ and MP), where a shorter 18S data for *C. labyrinthuloides* was used.

|  | **18S** | | | |  | ***rbc*L** | | | |
| --- | --- | --- | --- | --- | --- | --- | --- | --- | --- |
|  | ***S. grande*** | ***S. pusillum*** | ***Synchroma*** | **SCG clade** |  | ***S. grande*** | ***S. pusillum*** | ***Synchroma*** | **SCG clade** |
| ML no *G.w.* and no *C.s.* | 97 | 99 | 99 | 99 |  | 73 | 100 | 92 | 92 |
| ML no *G.w.* | 99 | 98 | 89 | 99 |  | 94 | 100 | --- ^2)^ | 79 |
| ML no *C.s.* | 99 | 98 | 97 | 99 |  | 91 | 87 | --- ^3)^ | 90 |
| ML all | **99** | **97** | **95** | **98** |  | **88** | **100** | **---** ^2) 3)^ | **85** |
| NJ no G.w. and no *C.s*. | 100 | 100 | 100 | 100 |  | 91 | 99 | 99 | 99 |
| NJ no *G.w.* | 100 | 95 | 68 | 99 |  | 99 | 99 | 84 | 87 |
| NJ no *C.s.* | 100 | 98 | 98 | 94 |  | 99 | 99 | --- ^2)^ | 99 |
| NJ all | **100** | **98** | **93** | **98** |  | **99** | **99** | **---** ^3)^ | **93** |
| MP no *G.w.* and no *C.s.* | 98 | 100 | 100 | 100 |  | 99 | 99 | 85 | 85 |
| MP no *G.w.* | 94 | 87 | 60 | 97 |  | 92 | 99 | 51 | 61 |
| MP no *C.s.* | 97 | 87 | 71 | 99 |  | 85 | 99 | --- ^3)^ | 93 |
| MP all | **98** | **92** | **72** | **94** |  | **91** | **99** | **---** ^3)^ | **---** |
| Bayesian no *G.w.* and no *C.s.* | 1.00 | 0.98 | 1.00 | 1.00 |  | 1.00 | 0.98 | 1.00 | 1.00 |
| Bayesian no *G.w.* | 0.98 (1.00) | 0.91 (1.00) | --- ^2)^ | 1.00 |  | 1.00 | 1.00 | --- ^2)^ | 1.00 |
| Bayesian no *C.s.* | 1.00 (1.00) | --- ^1)^ (0.96) | --- ^1)^ | 1.00 |  | 1.00 | 1.00 | --- ^3)^ | 1.00 |
| Bayesian all | **1.00 (1.00)** | **0.77 (1.00)** | **----** | **1.00** |  | **1.00** | **1.00** | **---** ^2) 3)^ | **1.00** |

--- split not present in consensus tree

^1)^ *S.p.* clade includes *G.w.*

^2)^ *Synchroma* clade includes *C.s.*

^3)^ *Synchroma* clade includes *G.w.*
