# Supplementary material for: Guanchochroma wildpretii gen. et spec. nov. (Ochrophyta) Provides New Insights into the Diversification and Evolution of the Algal Class Synchromophyceae
Source: PLoS One. 2015 Jul 2;10(7):e0131821. doi: 10.1371/journal.pone.0131821 (PMC4489749; doi:10.1371/journal.pone.0131821)
Supplement: S1 Text — (DOCX) [file pone.0131821.s015.docx]

**S1 Text. Phylogenetic analysis under compositional heterogeneity and rate variation across lineages.**

Since phylogenetic signal in a dataset can be superimposed with non-phylogenetic signal resulting from differing amounts of systematic error. These errors are caused by a variety of substitution model misspecifications, among others across-site rate variation, compositional heterogeneity and among-lineage rate variation (see summary in [1]), or simply saturation e.g. in 3^rd^ codon positions of protein-coding genes. An assessment of compositional homogeneity in datasets is enabled by the disparity index test [2], after which methods that subsequently cope with compositional bias in sequence alignments can be applied, such as LogDet transformation [3], a non-homogeneous (nh) substitution model [4] or RY-coding [5].

To account for compositional bias in the 18S rDNA dataset the data were (i) recoded to R (purine) and Y (pyrimidine) bases (RY coding, see e.g. [5]) and subjected to ML and Bayesian analysis. Bayesian Analysis was carried out for 5,000,000 generations as described for the 18S dataset above. ML analysis was carried out under raxmlGUI, with 10 runs of 100 bootstraps each, using a GTRCATI model [6]. The final 18S dataset was also (ii) LogDet transformed [3] and an optimal NJ tree calculated in MEGA 5.10 with different pattern among lineages under the pairwise deletion option for 1000 bootstraps. Another approach was (iii) the use of nhPhyML v0.1 [7] based on PhyML [8] to infer the ML phylogeny under a nonhomogeneous nonstationary substitution model according to [4]. For that purpose, default settings were extended to 10 gamma rate categories, optimized alpha parameter, 5 limited equilibrium frequencies and as the starting tree the ML tree obtained from the original 18S dataset calculation.

Also, long branch attraction (LBA) artefacts can occur under all inference methods (see review [9]) and datasets where unrelated taxa have a similar compositional bias or equally increased substitution are especially prone to it (see Class III long-branch effects, [10]). A simple method to test for LBA effects is the long-branch extraction, i.e. the observation of critical taxa positioning under removal of taxa with long branches [11]. All 18S calculations were therefore repeated under removal of either *C. socialis* or *G. wildpretii* (long-branch extraction).

1. Rodríguez-Ezpeleta N, Brinkmann H, Roure B, Lartillot N, Lang BF, Philippe H. Detecting and overcoming systematic errors in genome-sale phylogenies. Syst Biol. 2007;56: 389–399. doi:10.1080/10635150701397643

2. Kumar S, Gadagkar SR. Disparity Index: a simple statistic to measure and test the homogeneity of substitution patterns between molecular sequences. Genetics. 2001;158: 1321 –1327.

3. Lockhart PJ, Steel MA, Hendy MD, Penny D. Recovering evolutionary trees under a more realistic model of sequence evolution. Mol Biol Evol. 1994;11: 605–612.

4. Galtier N, Gouy M. Inferring pattern and process: Maximum-Likelihood implementation of a nonhomogeneous model of DNA sequence evolution for phylogenetic analysis. Mol Biol Evol. 1998;15: 871–879.

5. Phillips MJ, Penny D. The root of the mammalian tree inferred from whole mitochondrial genomes. Mol Phylogenet Evol. 2003;28: 171–185. doi:10.1016/S1055-7903(03)00057-5

6. Stamatakis A. Phylogenetic models of rate heterogeneity: a high performance computing perspective. Proc of IPDPS2006, Rhodos, Greece. 2006. doi:10.1109/IPDPS.2006.1639535

7. Boussau B, Gouy M. Efficient likelihood computations with nonreversible models of evolution. Syst Biol. 2006;55: 756–768. doi:10.1080/10635150600975218

8. Guindon S, Gascuel O. A simple, fast, and accurate algorithm to estimate large phylogenies by Maximum Likelihood. Syst Biol. 2003;52: 696–704. doi:10.1080/10635150390235520

9. Bergsten J. A review of long‐branch attraction. Cladistics. 2005;21: 163–193. doi:10.1111/j.1096-0031.2005.00059.x

10. Wägele JW, Mayer C. Visualizing differences in phylogenetic information content of alignments and distinction of three classes of long-branch effects. BMC Evol Biol. 2007;7: 147. doi:10.1186/1471-2148-7-147

11. Siddall ME, Whiting MF. Long-branch abstractions. Cladistics. 1999;15: 9–24. doi:10.1111/j.1096-0031.1999.tb00391.x
